# Does resistance training alone or in combination with aerobic training improve vascular function indices in adults with type 2 diabetes? A systematic review and meta-analysis of randomized controlled trials

**DOI:** 10.3389/fendo.2026.1824213

**Published:** 2026-05-15

**Authors:** Yanhao Wang, Yulong Wang, Ji Zhu, Wei Gao, Zhiheng Li, Xiaoyan Li, Ming Li

**Affiliations:** School of Physical Education and Sports Science, Fujian Normal University, Fuzhou, Fujian, China

**Keywords:** adults, aerobic training, meta-analysis, resistance training, systematic review, type 2 diabetes, vascular function

## Abstract

**Objective:**

To systematically evaluate and meta-analytically quantify the effects of RT-based interventions—defined as resistance training alone or resistance training combined with aerobic training—on vascular function in adults with T2DM.

**Methods:**

Following PRISMA guidelines, we systematically searched PubMed, Embase, Web of Science, the Cochrane Library, Ovid, CNKI, Wanfang Data, VIP, and CBM from inception to August 2025 for randomised controlled trials evaluating resistance training alone or combined with aerobic training on vascular function in adults with T2DM. Random-effects meta-analyses were conducted using *Hedge’ s g* and 95% confidence intervals (*CIs*). Heterogeneity was assessed with *I²*, and prespecified subgroup analyses and meta-regression were performed to explore potential moderators.

**Results:**

Compared with non-exercise controls, RT-based interventions significantly reduced arterial stiffness (*Hedge’ s g* = −0.24, *95% CI* −0.39 to −0.09; *p* = 0.0015) and improved endothelial function, as reflected by flow-mediated dilation (*Hedge’ s g* = 0.61, *95% CI* 0.32 to 0.89; *p* < 0.0001), in adults with T2DM. Subgroup analyses suggested that combined RT+AT generally produced more consistent benefits than RT alone, particularly in higher-intensity and longer-duration interventions, although meta-regression did not identify significant linear associations (*p* > 0.05). No significant effects were observed for wave reflection indices (*Hedge’ s g* = −0.10, *95% CI* −0.45 to 0.25; *p* = 0.58), and effects on peripheral haemodynamics remained inconclusive (*Hedge’ s g* = 0.44, *95% CI* −0.00 to 0.88; *p* = 0.05). These pooled findings should therefore be interpreted as reflecting RT-based interventions overall, rather than isolated RT per se.

**Conclusions:**

RT-based interventions, particularly when delivered as combined RT+AT, may improve vascular function in adults with T2DM, especially arterial stiffness and endothelial function, with moderate-certainty evidence supporting these benefits. However, because the pooled estimates reflect RT-based programmes overall and the evidence for RT alone was more limited for several outcomes, conclusions regarding isolated RT should remain cautious. Evidence for wave reflection indices remains inconclusive (moderate certainty), and evidence for peripheral haemodynamics remains inconclusive (low certainty). Further well-designed, adequately powered RCTs with standardised vascular assessments are needed to define optimal exercise prescriptions in adults with T2DM.

**Systematic review registration:**

https://www.crd.york.ac.uk/PROSPERO/, identifier CRD420261323648.

## 1 Introduction

Type 2 diabetes mellitus (T2DM) is a dominant driver of contemporary cardiometabolic disease burden and premature mortality, largely through its strong and pervasive association with cardiovascular disease (CVD) (1). Updated estimates from the 11th edition of the International Diabetes Federation (IDF) Diabetes Atlas indicate that 589 million adults aged 20–79 years were living with diabetes in 2024 (≈1 in 9), with projections approaching 853 million by 2050, underscoring the expanding population at risk for diabetes-related vascular complications (2). At the population level, large-scale pooled prospective evidence suggests that, at an adult diabetes prevalence of 10%, diabetes may account for approximately 11% (10–12%) of vascular deaths, highlighting the substantial cardiovascular mortality burden attributable to dysglycaemia (1). Beyond conventional risk factors, vascular dysfunction is increasingly viewed as a central pathophysiological feature of T2DM and a plausible mediator linking metabolic dysregulation to clinical cardiovascular events (3). Chronic hyperglycaemia and insulin resistance—through oxidative stress, low-grade inflammation, and advanced glycation pathways—can impair endothelial signalling and promote adverse vascular remodelling (4). Consistent with these mechanisms, adults with T2DM frequently exhibit impaired endothelium-dependent vasodilation, increased arterial stiffness, and perturbed wave reflection, each of which is clinically aligned with heightened CVD risk and poorer prognosis (5–7).

Vascular function indices offer clinically meaningful, noninvasive intermediate endpoints that capture complementary levels of the arterial tree and can be used to evaluate the vascular impact of lifestyle and pharmacological interventions (8). Conduit artery endothelial function is commonly quantified using flow-mediated dilation (FMD), while large-artery stiffness is typically assessed using pulse wave velocity (PWV), with carotid–femoral PWV considered a gold-standard measure and an important biomarker for cardiovascular risk assessment (9–11). Wave reflection indices (e.g., augmentation index [AIx]) provide additional information on arterial tone and the timing/magnitude of reflected pressure waves (12), whereas peripheral haemodynamic measures (e.g., blood flow and systemic vascular resistance) help characterize resistance vessel function and microvascular control (13). Importantly, these intermediate phenotypes are not only physiologically informative but also clinically relevant: meta-analytic evidence from observational cohorts indicates that brachial FMD is associated with future cardiovascular events, supporting its use as a surrogate vascular marker in intervention research (14). Collectively, a multidomain vascular assessment (stiffness, endothelial function, wave reflection, peripheral haemodynamics) may therefore provide a more complete characterization of vascular dysfunction in T2DM and a more sensitive evaluation of exercise-mediated vascular adaptations.

Exercise training is a cornerstone of non-pharmacological management in T2DM, with established benefits for glycaemic control, body composition, cardiorespiratory fitness, and several CVD risk factors (15, 16). Aerobic training has historically been emphasized for vascular health, in part because repeated elevations in shear stress during endurance exercise may enhance endothelial function (17). RT, however, is increasingly recognized as an essential component of T2DM exercise prescription owing to its benefits for skeletal muscle mass and strength, insulin sensitivity, and functional capacity—adaptations that may facilitate long-term adherence and metabolic control (18, 19). From a vascular perspective, plausible mechanisms by which RT could influence vascular function include changes in peripheral vascular conductance and blood pressure regulation, improvements in endothelial responsiveness, and structural or functional adaptations within the arterial wall (20). Nevertheless, the direction and magnitude of vascular adaptations to RT in T2DM may not be uniform across vascular domains, and the trial literature has reported variable findings, including across different RT intensities (21, 22).

Indeed, the extent to which RT alone—and RT combined with aerobic training (RT+AT)—improves vascular function in adults with T2DM remains uncertain (23–25). Randomized controlled trials have shown heterogeneous effects across outcomes such as arterial stiffness and endothelial function (26–28), and such inconsistency may reflect differences in participant profiles (e.g., age, sex composition, baseline vascular status) as well as differences in exercise prescription (e.g., modality, intensity, frequency, session duration, and program length, alongside RT dose descriptors such as sets and repetitions) (25). Moreover, RT-based interventions implemented in clinical and community settings are diverse, spanning machine/free-weight programs (26–28), elastic-band RT (29), and structured bodyweight “resistance-type” activities used to interrupt prolonged sitting—approaches that may elicit distinct haemodynamic and vascular stimuli and, consequently, divergent vascular responses (30). A focused synthesis that isolates RT-based interventions while evaluating multiple vascular domains is therefore required to inform evidence-based exercise prescription for vascular health in T2DM (23, 24).

Previous systematic reviews in this area have often prioritized glycaemic or broad cardiometabolic outcomes; when vascular outcomes were considered, synthesis was commonly restricted to a single domain (e.g., endothelial function or arterial stiffness) and/or pooled heterogeneous exercise modalities without isolating RT-based approaches (31–33). When vascular endpoints were explicitly addressed, the focus frequently remained on endothelial function indexed by brachial FMD (or on concurrent training as a combined modality), rather than providing a RT-centric, multi-domain vascular synthesis in adults with T2DM (34–36). In addition, potential moderators related to participant characteristics and exercise dose have not consistently been examined using pre-specified subgroup analyses and meta-regression, limiting translation into actionable training prescriptions. Against this backdrop, there is a clear need to (i) quantify the vascular effects of RT alone versus RT+AT, (ii) synthesize evidence across multiple vascular domains (arterial stiffness, endothelial function, wave reflection, and peripheral haemodynamics), and (iii) evaluate whether key participant and program characteristics explain between-study variability and inform optimization of RT-based prescriptions in adults with T2DM.

Because both RT alone and RT combined with aerobic training retain a resistance-training stimulus, the present review treated them together as RT-based interventions at the primary synthesis level. However, given their potentially different physiological effects on vascular adaptation, intervention modality (RT alone vs RT+AT) was examined as a prespecified moderator to clarify whether pooled findings primarily reflected isolated RT or the broader effect of multicomponent programmes that include RT. Therefore, the primary aim of this systematic review and meta-analysis of randomized controlled trials was to quantify the effects of RT alone or RT combined with aerobic training, compared with non-exercise/usual care controls, on vascular function outcomes in adults with T2DM. Outcomes of interest encompassed arterial stiffness (e.g., PWV/cfPWV and related stiffness indices), endothelial function (FMD), wave reflection and peripheral haemodynamic measures (e.g., blood flow and systemic vascular resistance). The secondary aim was to examine whether participant characteristics and intervention parameters (modality, intensity, frequency, session duration, program length, and RT dose descriptors such as sets and repetitions) moderated intervention effects, thereby helping to explain heterogeneity and providing clinically actionable evidence to optimize RT-based exercise prescription for vascular health in T2DM.

## 2 Methods

This systematic review was conducted in accordance with the Preferred Reporting Items for Systematic Reviews and Meta-Analyses (PRISMA) guidelines (37), and was registered retrospectively in PROSPERO (registration number: CRD420261323648).

### 2.1 Information sources

We systematically searched PubMed, Embase, Web of Science, the Cochrane Library, Ovid, China National Knowledge Infrastructure (CNKI), Wanfang Data, VIP, and the Chinese Biomedical Literature Database (CBM) from inception to August 2025. No restrictions were applied with respect to publication year, publication type, or language.

To enhance coverage beyond the electronic searches, we applied three complementary snowballing strategies: (1) screening the reference lists of all included studies; (2) identifying and assessing studies that cited the included articles; and (3) expanding retrieval using MEDLINE’s “Similar articles” function and Embase’s “Find similar” feature to capture additional potentially eligible records. All searches were completed in August 2025.

### 2.2 Literature search strategy

The search strategy was informed by previous systematic reviews on related topics, and the search terms included: (“resistance training”[MeSH Terms] OR (“resistance”[All Fields] AND “training”[All Fields]) OR “resistance training”[All Fields]) AND ((“blood vessels”[MeSH Terms] OR (“blood”[All Fields] AND “vessels”[All Fields]) OR “blood vessels”[All Fields] OR “vascular”[All Fields] OR “neovascularisation, pathologic”[MeSH Terms] OR (“neovascularisation”[All Fields] AND “pathologic”[All Fields]) OR “pathologic neovascularisation”[All Fields] OR “vascularisation”[All Fields] OR “vascularisation”[All Fields] OR “vascularisations”[All Fields] OR “vascularise”[All Fields] OR “vascularised”[All Fields] OR “vascularities”[All Fields] OR “vascularitis”[All Fields] OR “vascularity”[All Fields] OR “vascularisations”[All Fields] OR “vascularise”[All Fields] OR “vascularised”[All Fields] OR “vascularises”[All Fields] OR “vascularising”[All Fields] OR “vasculars”[All Fields]) AND (“functional”[All Fields] OR “functional s”[All Fields] OR “functionalities”[All Fields] OR “functionality”[All Fields] OR “functionalisation”[All Fields] OR “functionalisations”[All Fields] OR “functionalise”[All Fields] OR “functionalised”[All Fields] OR “functionalises”[All Fields] OR “functionalising”[All Fields] OR “functionally”[All Fields] OR “functionals”[All Fields] OR “functioned”[All Fields] OR “functioning”[All Fields] OR “functionings”[All Fields] OR “functions”[All Fields] OR “physiology”[MeSH Subheading] OR “physiology”[All Fields] OR “function”[All Fields] OR “physiology”[MeSH Terms])).

### 2.3 Selection process

All records retrieved from the database searches were imported into EndNote 21, and duplicates were removed manually by one reviewer (Y.-H.W.). Two reviewers (Y.-L.W. and W.G.) independently screened titles and abstracts against the prespecified inclusion and exclusion criteria. Records that were clearly irrelevant to the review question were excluded at this stage. Disagreements were resolved by discussion; if consensus could not be reached, a third reviewer (Z.-H.L.) adjudicated.

Full texts were then sought for all potentially eligible reports. When full texts could not be obtained despite repeated attempts through database/platform retrieval, manual searching, and contact with corresponding authors, these reports were classified as “not retrieved.” The same two reviewers then independently assessed all retrieved full-text articles for eligibility and documented reasons for exclusion at the full-text stage. Any remaining discrepancies were again resolved through discussion, with arbitration by a third reviewer when required. In addition to the database searches, we examined relevant systematic reviews and screened the reference lists of included studies; drawing on the research team’s domain expertise, we also sought to identify potentially eligible trials that may not have been captured by the initial search strategy. The study selection process is summarised using a PRISMA flow diagram.

### 2.4 Eligibility criteria and exclusion criteria

Eligibility criteria were prespecified according to the PICOS framework. We included randomised controlled trials enrolling adults (≥ 18 years) with type 2 diabetes mellitus. Interventions were required to include resistance training, delivered either as a standalone modality or in combination with aerobic training, with no restrictions on delivery format (e.g., machine-based or free-weight programmes, elastic-band training, or structured bodyweight resistance exercise). Eligible comparators were non-exercise/usual-care control conditions without structured exercise training. Trials were included if they reported at least one vascular-function outcome, encompassing indices of arterial stiffness (e.g., pulse wave velocity, carotid–femoral pulse wave velocity, or the β-stiffness index), endothelial function (e.g., flow-mediated dilation), wave reflection (e.g., augmentation index), or peripheral haemodynamics (e.g., blood flow or systemic vascular resistance).

Studies were excluded if they met any of the following criteria: (1) non-randomised designs; (2) participants without T2DM and/or not adults; (3) interventions that did not include a resistance-training component, or multi-component programmes in which the independent effect of resistance training could not be reasonably disentangled; (4) absence of an eligible non-exercise/usual-care control group; or (5) failure to report eligible vascular-function outcomes, or insufficient data to permit effect-size estimation. Duplicate reports from the same trial were also excluded, with only the most complete dataset retained.

### 2.5 Data extraction and transformation

Data extraction was performed independently by two reviewers (Y.-H.W. and Y.-L.W.) using a standardised extraction form that was developed in Microsoft Excel and pilot-tested before formal use. For each eligible study, we systematically extracted and cross-checked the following information: (1) study characteristics (first author, publication year, country/region, journal, and study design); (2) participant characteristics (sample size, mean age, and sex distribution); (3) intervention characteristics (modality [RT alone vs RT+AT], programme duration, weekly frequency, session duration, intensity, repetitions, sets, supervision, progression, total intervention sessions, and, where available, achieved dose/adherence); (4) outcome data (vascular-function outcomes, including arterial stiffness, endothelial function, and other vascular indices; measurement/assessment methods; statistical parameters required for effect-size computation; and key findings); and (5) when a single publication reported multiple independent studies or multiple comparisons (e.g., different training prescriptions or intervention arms), data were extracted separately for each study/comparison arm to preserve the independence of effect-size estimates.

When data were missing, incompletely reported, or ambiguously described, we prioritised contacting the corresponding authors to obtain additional information. If the required data could not be retrieved, we applied established statistical conversion methods—where methodologically appropriate—to transform the reported summary statistics into formats suitable for pooling (e.g., means and standard deviations, or pre–post change scores with corresponding measures of variability), thereby meeting the requirements for meta-analytic synthesis. Where intervention-prescription details were incompletely or inconsistently reported across studies, they were described narratively and interpreted cautiously in relation to moderator analyses.

### 2.6 Data extraction

Where studies reported confidence intervals (CI), these were converted to standard deviations (SD) (38):


$$SD=\sqrt{N}\frac{CI_{\text{hugh}}-CI_{low}}{2t}$$

Where studies reported standard errors (SE), these were converted to SD (38):


$$SD=\sqrt{N}\times SE$$

Baseline and final time-point values for vascular function outcomes were extracted from each study, and the mean pre–post change (mean difference, MD) was calculated using the following formula (38):


$$MD_{diff}=M_{post}-M_{pre}$$

The SD of the pre–post change scores was calculated using the following formula (38):


$$SD_{\text{diff}}=\sqrt{SD_{\text{pre}}^{2}+SD_{\text{post}}^{2}-2r\times SD_{\text{pre}}\times SD_{\text{post}}}$$

In accordance with the recommendations of the Cochrane Handbook, we contacted the corresponding authors of the included trials to obtain the correlation coefficients (r values) required for pre–post data analyses.

### 2.7 Risk of bias and quality of methods assessment

Risk of bias was independently assessed by two reviewers (Y.-H.W. and J.Z.). Discrepancies were first addressed through detailed discussion; if consensus could not be reached, an additional reviewer arbitrated and made the final judgement.

We evaluated each included study using the Cochrane risk-of-bias tool across seven prespecified domains: (1) random sequence generation; (2) allocation concealment; (3) blinding of participants and personnel; (4) blinding of outcome assessors; (5) completeness of outcome data; (6) selective outcome reporting; and (7) other potential sources of bias.

For blinding, we evaluated blinding procedures separately for participants, intervention personnel (e.g., exercise supervisors), and outcome assessors. Given the inherently recognisable nature of exercise-training interventions, blinding of participants and intervention personnel is often not feasible in practice; therefore, the absence of blinding in these two domains was not used as a prespecified criterion for study inclusion or exclusion. In contrast, judgements regarding blinding of outcome assessment were primarily based on whether vascular outcomes were obtained using objective, standardised assessment procedures (e.g., instrument-based measurements).

Each domain was rated as “low risk”, “unclear risk”, or “high risk” of bias. Based on the pattern of judgements across the seven domains, an overall risk-of-bias rating (low, moderate, or high) was assigned to each study. Risk-of-bias summary tables and figures were generated using Review Manager (RevMan) version 5.4.1.

### 2.8 Statistical analysis

All statistical analyses and data visualisations were conducted in R (version 4.5.0), primarily using the meta, metafor, and metameta packages. Pooled effect estimates were synthesised using random-effects models and summarised with inverse-variance weighting employing the DerSimonian–Laird method (39). Between-study heterogeneity (τ²) and its confidence interval were estimated using the Jackson method. Depending on the information reported in the included trials, we extracted or derived mean differences (MDs) or standardised mean differences (SMDs), together with their 95% confidence intervals. Given the substantial variability in measurement units across vascular outcomes, and in line with methodological recommendations, effect estimates were harmonised using SMDs where appropriate to enable valid pooling across studies (40), Standardised mean differences (SMDs) were used as the primary effect metric. Given that most included trials had small sample sizes (*n* < 50), we applied *Hedge’s g* to correct for small-sample bias; hereafter, effect sizes are reported as g. For clinical interpretation, we used conventional thresholds: 0–0.2 (trivial), 0.2–0.5 (small), 0.5–0.8 (moderate), and > 0.8 (large) (41). To evaluate the robustness of both the overall and subgroup effect estimates, and to reduce the risk of type II error attributable to limited statistical power, we additionally conducted statistical power analyses (42).

Between-study heterogeneity was assessed comprehensively using the Cochrane Q test, the I² statistic, and heterogeneity variance and standard deviation estimates (τ² and τ), in accordance with current methodological guidance (40). In particular, the magnitude of heterogeneity was primarily interpreted using I² thresholds: 0–25% (low), 25–75% (moderate), and > 75% (high) (43). We also calculated prediction intervals to characterise the plausible range of true effects across different study contexts and to appraise the external applicability of the pooled estimates.

The primary meta-analyses pooled all RT-based interventions versus non-exercise/usual-care controls because all eligible interventions contained a resistance-training component. To address the possibility that RT alone and RT+AT may differ in their vascular effects, intervention modality was examined *a priori* as a subgroup factor and interpreted explicitly in the results and discussion. Subgroup analyses and meta-regression were conducted strictly in accordance with the preregistered protocol, focusing on two categories of potential moderators: participant characteristics and intervention-prescription features. Categorical variables were examined using prespecified subgroup analyses, whereas continuous variables were evaluated using mixed-effects meta-regression models (44). Model parameters were estimated using restricted maximum likelihood (REML). The final functional form was selected by comparing the bias-corrected goodness-of-fit of linear specifications with that of cubic spline functions. Relative to conventional maximum-likelihood estimation, this approach is generally considered more robust in mixed-effects frameworks that simultaneously incorporate fixed and random effects (45).

Publication bias and small-study effects were assessed using visual inspection of funnel plots and Egger’s linear regression test (46, 47). When Egger’s test yielded *p* > 0.05, we considered there to be no clear evidence of substantial publication bias. All statistical tests were two-sided; *p* < 0.05 was regarded as statistically significant, whereas 0.05 ≤ *p* < 0.10 was interpreted as indicating a potential statistical trend.

### 2.9 Certainty of the evidence

We used the GRADE framework to rate the certainty of evidence for the primary outcomes, classifying it as high, moderate, low, or very low. Certainty ratings were determined by considering the following key domains for potential downgrading:

(1) Risk of bias: if the overall risk of bias for a given outcome was judged to raise “some concerns”, the certainty of evidence was downgraded by one level; if the overall judgement indicated a high risk of bias, certainty was downgraded by two levels. (2) Inconsistency: certainty was downgraded by one level when between-study heterogeneity was moderate and by two levels when heterogeneity was substantial. (3) Imprecision: certainty was downgraded by one level when effect estimates were clearly imprecise (e.g., the 95% confidence interval crossed the line of no effect and the pooled result was not statistically significant). (4) Publication bias: certainty was downgraded by one level when Egger’s linear regression suggested small-study effects or a potential risk of publication bias.

All GRADE assessments were completed independently by two authors (the first and third authors). Any discrepancies were resolved through discussion to maximise the objectivity and transparency of the evidence-certainty rating process.

## 3 Results

### 3.1 Literature search results

**Figure 1:**
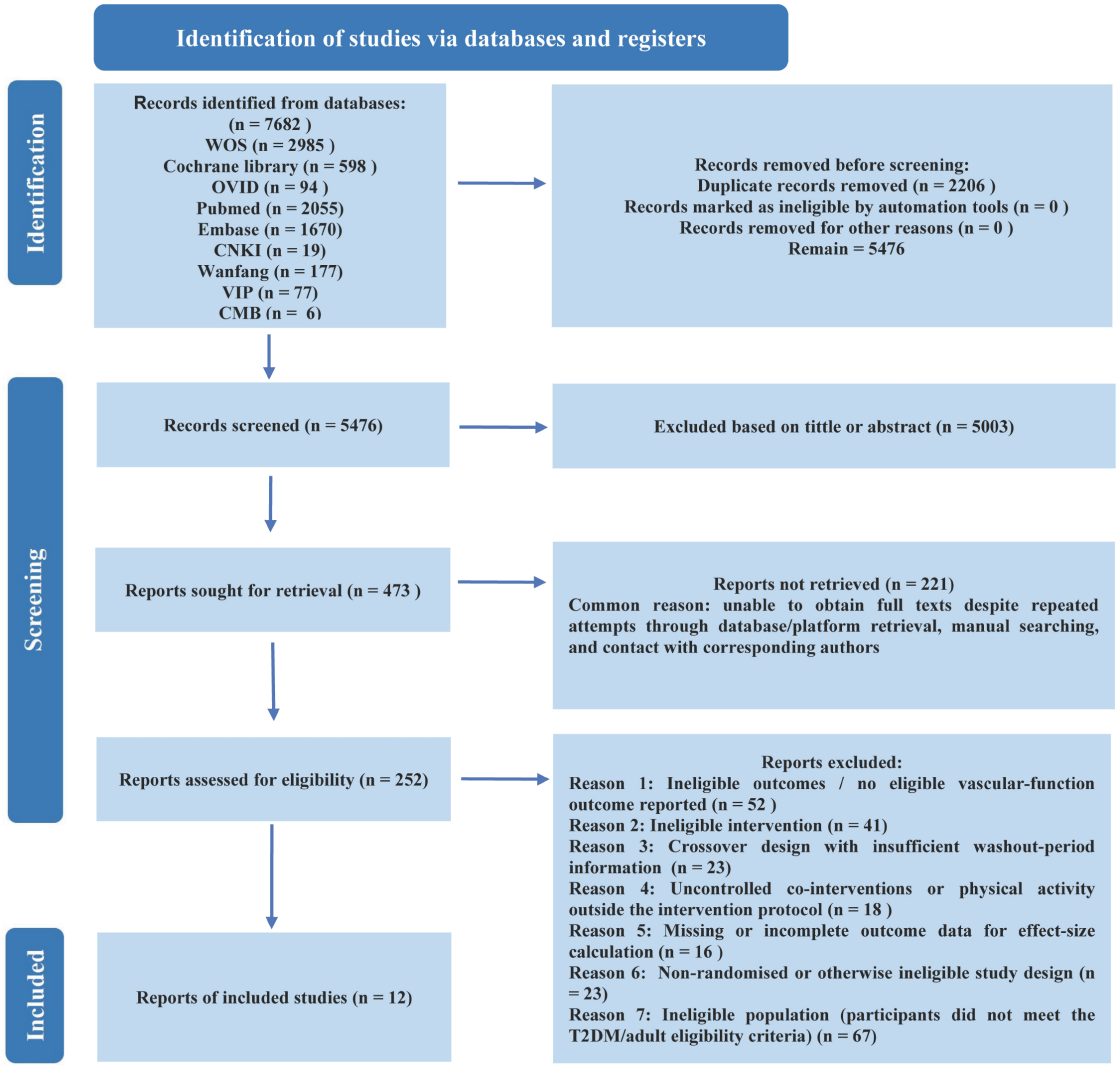
Flow diagram of literature selection.

The database searches yielded 7,682 records. After duplicate removal, 5,476 records underwent title/abstract screening, of which 5,003 were excluded as clearly irrelevant to the review question. Full texts were sought for 473 reports; 221 could not be retrieved despite repeated retrieval attempts, and 252 full-text articles were assessed for eligibility. After full-text assessment, 240 reports were excluded with reasons, and 12 studies met the inclusion criteria and were included in the systematic review and meta-analysis (**Figure 1**).

### 3.2 Analysis of basic characteristics of included studies

As shown in **Table 1**, this review included 12 randomised controlled trials. Most trials enrolled adults with T2DM, although a minority included individuals who were overweight or obese with concomitant T2DM; all comparators were non-exercise/usual-care controls. Sample sizes ranged from approximately 13 to 140 participants per trial, and mean ages spanned roughly 18–63 years. Sex composition varied across studies, indicating some heterogeneity in population representativeness. Interventions broadly comprised resistance training alone (RT), combined aerobic and resistance training (RT+AT), and short-duration resistance-type activity protocols using predominantly bodyweight movements. Prescription characteristics varied substantially across trials, particularly in intensity, frequency, session duration, and programme length. Most studies appeared to involve at least an initial supervised component, although supervision was often described only broadly. Outcomes included arterial stiffness, FMD, wave reflection, and peripheral haemodynamics, but the combinations reported were inconsistent across studies. Importantly, most included comparisons involved RT+AT, whereas RT-alone evidence was comparatively limited, particularly for several secondary vascular outcomes. Reporting completeness also varied across exercise-prescription variables: modality, weekly frequency, session duration, and programme duration were available for most interventions, whereas repetitions, sets, supervision, progression, achieved dose, and adherence were reported less consistently. Accordingly, moderator findings should be interpreted cautiously.

**Table 1:** Characteristics of included studies.

Study	Health status	Age(years)	Participants(M/F)		Intervention		Description of intervention	Intensity	Repetitions	Sets	Frequency(t/wk)	Time(min)	Duration(wk)	Outcomet
		Intervention/Control	Intervention	Control	Intervention	Control								
McGavok et al., 2004 (48)	T2DM	59	0/11	0/7	AT+RT	non-exercise	Supervised combined aerobic cycling plus machine-based resistance training for major upper- and lower-body muscle groups.	AT:65–75% HRR RT:50–65% 1RM High intensity	10-15	3	3	NR	10	SVR
Taylor et al., 2020 (30)	T2DM	61.5	13/11	13/11	SRA3	non-exercise	Brief bodyweight resistance-type activity breaks used to interrupt prolonged sitting, including calf raises, knee raises, and sit-to-stand movements.	Light intensity (bodyweight)	NR	NR	NR	3	1	FMD, BF
					SRA6		Brief bodyweight resistance-type activity breaks using the same movements as SRA3, delivered in a longer interruption format.					6		
Kwon et al., 2011 (49)	Overweight/T2DM	58	0/12	0/15	RT	non-exercise	Supervised elastic-band resistance training targeting upper-body, lower-body, and core muscle groups, with progressive loading.	40-50% 1RM Low intensity	10-15	3	3	40	12	FMD
Okada et al., 2010 (50)	T2DM	63	10/11	11/6	AT+RT	non-exercise	Supervised combined aerobic dance, stationary cycling, and resistance training programme.	Training heart rate determined by Karvonen’s equation (k=0.6) High intensity	NR	NR	3-5	75	12	FMD
Dobrosielski et al., 2012 (51)	T2DM	57	41/29	40/30	AT+RT	non-exercise	Supervised combined aerobic exercise (treadmill, cycle ergometer, or stepper) plus resistance training for major muscle groups.	AT:60–90% HRmax RT:≈50%1RM High intensity	10-15	2	3	45	24	cfPWV
Loimaala et al., 2009 (27)	T2DM	54	24/0	24/0	AT+RT	non-exercise	Long-term combined endurance and strength training using walking/jogging plus machine- and free-weight resistance exercises for major muscle groups.	AT:65–75% VO_2_max RT:60–80% MVC High intensity	8-12	3	4	≥30	104	PWV
Loimaala et al., 2003 (52)	T2DM	54	24/0	25/0	AT+RT	non-exercise	Combined endurance training and circuit-based strength training targeting trunk and upper- and lower-limb muscle groups.	AT:65–75% VO_2_max RT:70–80% MVC High intensity	10-12	3	4	30	52	PWV
Maiorana et al., 2001 (26)	T2DM	52	14/2	14/2	AT+RT	non-exercise	Circuit-based combined aerobic and resistance training alternating cycle ergometer work with resistance exercises.	AT:70–85% HRpeak RT:55–65% MVC High intensity	to failure	NR	3	60	8	FMD
Naylor et al., 2016 (53)	T2DM	18	2/6	1/4	AT+RT	non-exercise	Supervised gym-based combined aerobic and multi-joint resistance training with additional core and stretching components.	AT:65–85% HRmax RT:55–70% MVC High intensity	NR	NR	3	60	12	FMD
Russell et al., 2017 (54)	T2DM	52	11/6	11/6	RT	non-exercise	Supervised whole-body resistance training using free weights and machines, with additional core and stability sessions.	RT:65–85% 1RM High intensity	6-15	1	3	60	6	PWV, AIx
Cox et al., 2024 (28)	T2DM	59	14/9	14/9	AT+RT	non-exercise	Supervised low-volume combined aerobic and resistance high-intensity interval training, followed by self-directed continuation.	AT:50–60% HRpeak RT: RPE≥ 17 High intensity	10-25	8	3	26	8	FMD, cfPWV, AIx,
		60	14/9	14/9	AT+RT		Supervised combined moderate-intensity aerobic and resistance training, followed by self-directed continuation.	AT:55–69% HRpeak RT: RPE 11–13 moderate-intensity	10	2	4	52.5		
Magalhães et al., 2019 (55)	T2DM	59	15/13	13/14	AT+RT	non-exercise	Supervised combined moderate-intensity continuous cycling plus whole-body resistance training.	AT:40–60% HRR RT:10–12RM High intensity	10-12	1	3	40-60	52	cfPWV, cdPWV, crPWV, β-stiffness index
		58	10/15	13/14	AT+RT		Supervised combined interval cycling plus whole-body resistance training with matched weekly energy expenditure.	AT:70–90% HRR RT:10–12RM High intensity	10-12	1	3	30-45		

T2DM, type 2 diabetes mellitus; RT, Resistance Training; AT, Aerobic Training; SRA3 or 6, Simple Resistance Activities; PWV, Pulse Wave Velocity; cfPWV, Carotid–Femoral Pulse Wave Velocity; cdPWV, carotid to distal posterior tibial artery pulse wave velocity; crPWV, carotid to radial artery pulse wave velocity; FMD, flow-mediated dilation; PVR, peripheral vascular resistance; SVR, systemic vascular resistance; BF, blood flow; AIx, augmentation index; NR, not reported.

### 3.3 Results of risk of bias assessment

**Figure 2:**
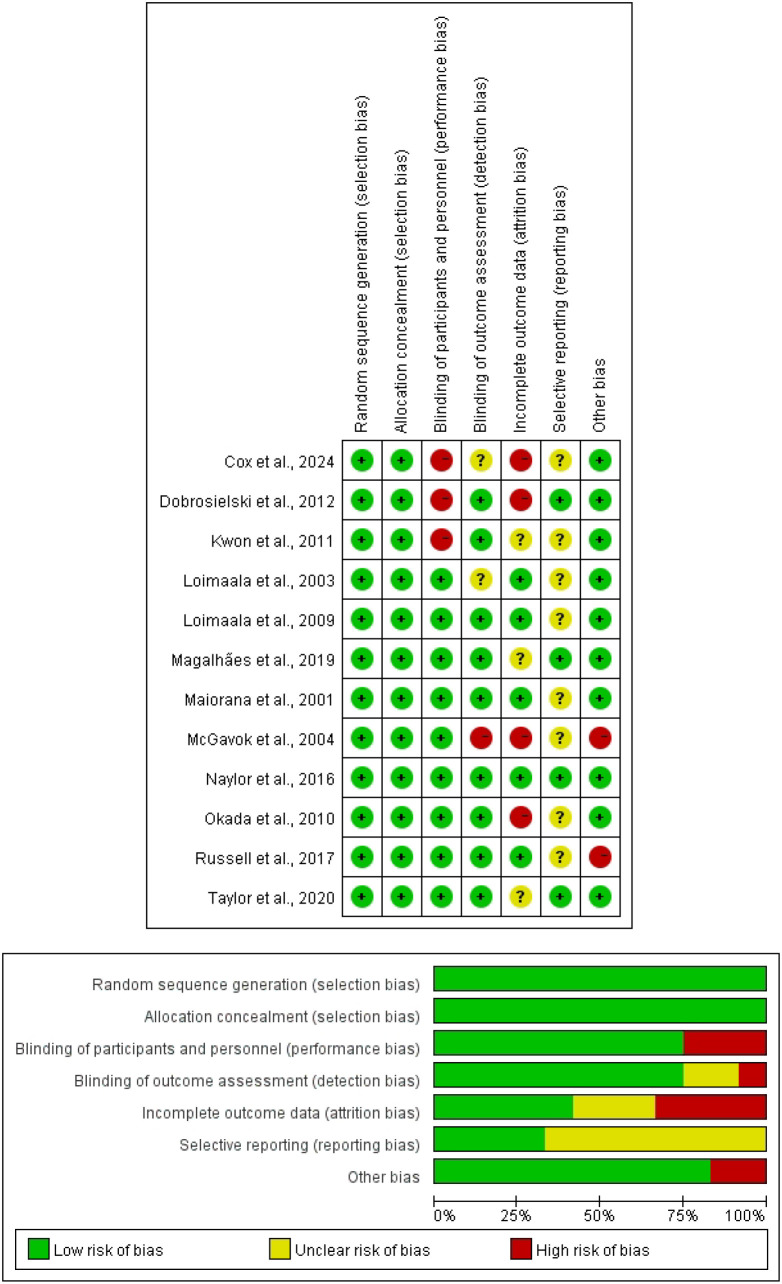
Risk of bias results.

We systematically appraised the methodological quality of the included trials using the Cochrane risk-of-bias tool, and generated risk-of-bias summary plots with Review Manager (RevMan 5.4.1). Overall, the predominant risks of bias were concentrated in three domains—randomisation-related procedures, outcome measurement, and selective outcome reporting—each of which may compromise the reliability of the findings and introduce potential bias (**Figure 2**).

### 3.4 Results of meta-analysis

Only intervention variables with sufficient and reasonably consistent reporting across studies were entered into subgroup analyses or meta-regression; supervision, progression strategy, and achieved dose/adherence could not be examined quantitatively because reporting was sparse or non-standardised.

#### 3.4.1 Effects of resistance training alone or combined with aerobic training on arterial stiffness in adults with type 2 diabetes

**Figure 3:**
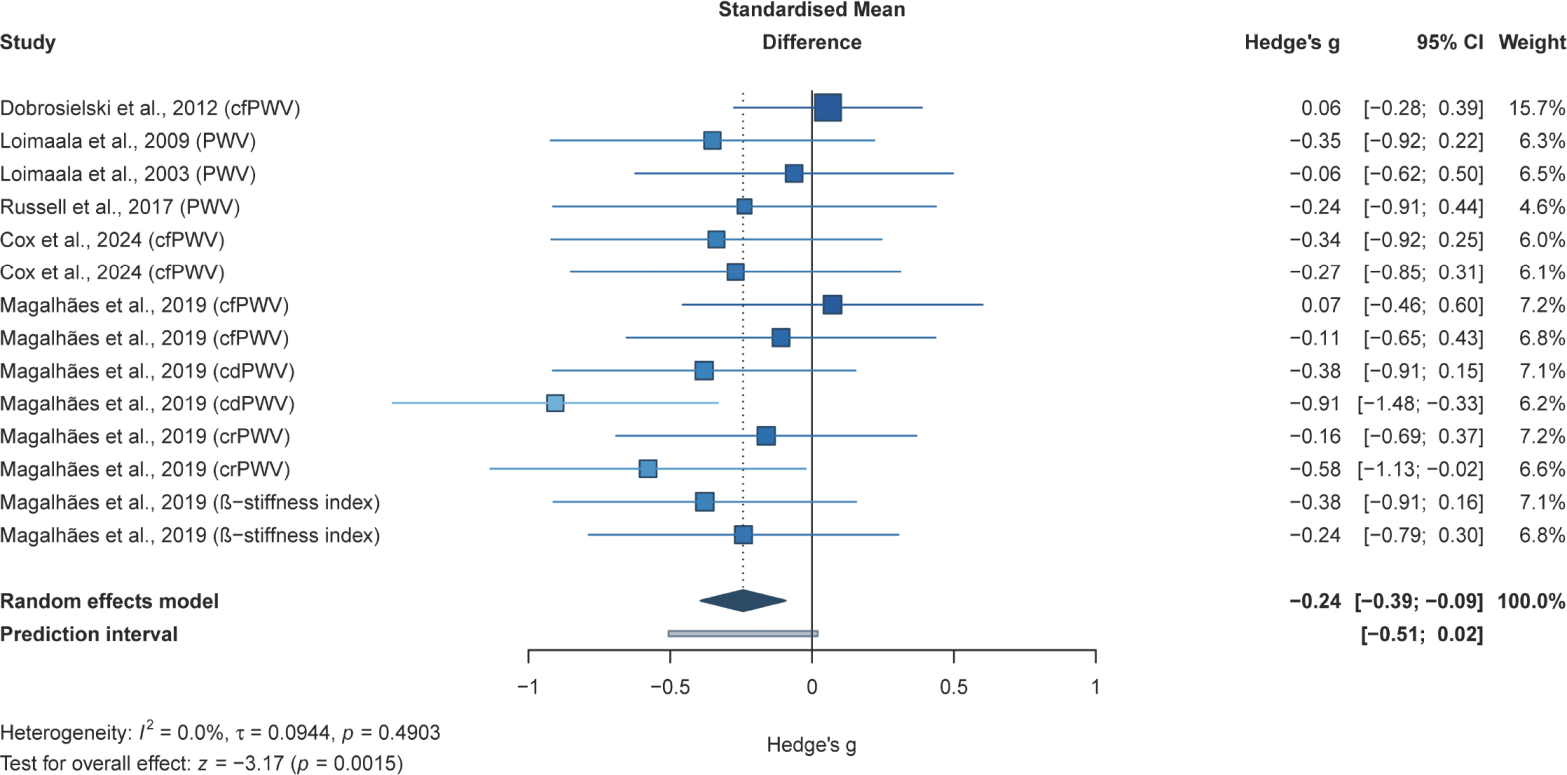
Forest plot for arterial stiffness. Forest plot of the effects of resistance training alone or resistance training combined with aerobic training on arterial stiffness in adults with T2DM, compared with non-exercise/usual-care controls; 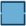, squares represent study-specific effect estimates, with square area proportional to study weight; 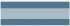, horizontal lines indicate 95% confidence intervals; 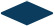, The diamond represents the pooled effect estimate, and its width indicates the 95% CI. Negative values favour exercise for arterial stiffness outcomes.

The meta-analysis of six studies (14 effect sizes; 791 participants) suggested that, compared with non-exercise controls, RT-based interventions may be associated with a small reduction in arterial stiffness in adults with T2DM (*g* = −0.24, *95% CI* −0.39 to −0.09; *p* = 0.0015; **Figure 3**).

Subgroup analyses for this outcome were conducted according to participant characteristics and intervention-prescription features, with subgroup pooled effects estimated using the same random-effects model as in the primary analysis. The findings appeared to suggest possible between-subgroup differences for sex, arterial stiffness measure type, intervention modality, repetitions, sets, frequency, session duration, and programme duration (*p* < 0.05; **Table 2**). However, these patterns should be interpreted cautiously because several strata were informed by few studies and the analyses were exploratory.

**Table 2:** Subgroup analysis of meta-analysis results of arterial stiffness.

Subgroup		K(N)	Hedges’g	95% CI	P _d_	Q	I ^2^ (%)	Power(%)	P _m_
Age									*N/A*
	Young Adult: 19–24	*N/A*	*N/A*	*N/A*	*N/A*	*N/A*	*N/A*	*N/A*	
	Adults aged 25–44	*N/A*	*N/A*	*N/A*	*N/A*	*N/A*	*N/A*	*N/A*	
	Middle Aged: 45–64	14(791)	-0.24	-0.39 to -0.09	0.0015	12.46	0	10	
	Aged: ≥ 65	*N/A*	*N/A*	*N/A*	*N/A*	*N/A*	*N/A*	*N/A*	
Sex									0.001
	Male	2(97)	-0.20	-0.60 to 0.19	0.31	0.49	0	10	
	Female	*N/A*	*N/A*	*N/A*	*N/A*	*N/A*	*N/A*	*N/A*	
	Mixed-sex	12(694)	-0.25	-0.42 to -0.09	0.0031	11.93	7.9	14.38	
Arterial stiffness measure type									0.009
	PWV	3(131)	-0.21	-0.56 to 0.13	0.22	0.5	0	10	
	cfPWV	5(339)	-0.06	-0.28 to 0.15	0.55	2.11	0	10	
	cdPWV	2(107)	-0.63	-1.15 to -0.12	0.01	1.72	42	21.97	
	crPWV	2(107)	-0.36	-0.77 to 0.05	0.08	1.13	11.5	12.2	
	β-stiffness index	2(105)	-0.31	-0.69 to 0.07	0.1	0.12	0	10	
Intervention									0.0015
	RT	1(34)	-0.24	-0.91 to 0.44	*N/A*	*N/A*	*N/A*	*N/A*	
	RT+AT	13(757)	-0.25	-0.40 to -0.09	0.0021	12.46	3.7	11.97	
Intensity									*N/A*
	Bodyweight	*N/A*	*N/A*	*N/A*	*N/A*	*N/A*	*N/A*	*N/A*	
	Low intensity	*N/A*	*N/A*	*N/A*	*N/A*	*N/A*	*N/A*	*N/A*	
	Moderate intensity	*N/A*	*N/A*	*N/A*	*N/A*	*N/A*	*N/A*	*N/A*	
	High intensity	14(791)	-0.24	-0.39 to -0.09	0.0015	12.46	0	10	
	To failure	*N/A*	*N/A*	*N/A*	*N/A*	*N/A*	*N/A*	*N/A*	
Repetitions									0.0006
	6-15	14(791)	-0.24	-0.39 to -0.09	0.0015	12.46	0	10	
	16-25	2(92)	-0.30	-0.71 to 0.11	0.14	0.025	0	10	
Sets									0.02
	1	9(462)	-0.32	-0.50 to -0.13	0.0008	8.08	1.1	10.46	
	2	1(140)	0.06	-0.28 to 0.39	*N/A*	*N/A*	*N/A*	*N/A*	
	3	2(97)	-0.20	-0.60 to 0.19	0.31	0.49	0	10	
	8	2(92)	-0.30	-0.71 to 0.11	0.14	0.025	0	10	
Frequency(t/wk)									0.0018
	3	12(266)	-0.25	-0.42 to -0.09	0.003	11.93	7.9	14.38	
	4	2(97)	-0.20	-0.60 to 0.19	0.31	0.49	0	10	
Time(min)									0.0002
	26	1(46)	-0.34	-0.92 to 0.25	*N/A*	*N/A*	*N/A*	*N/A*	
	30-45	11(665)	-0.24	-0.42 to -0.07	0.0072	12.31	18.8	22.28	
	45-60	7(348)	-0.24	-0.45 to -0.03	0.02	2.10	0	10	
Duration(wk)									0.01
	6	1(34)	-0.24	-0.91 to 0.44	*N/A*	*N/A*	*N/A*	*N/A*	
	8	2(92)	-0.30	-0.71 to 0.11	0.14	0.025	0	10	
	24	1(140)	0.06	-0.28 to 0.39	*N/A*	*N/A*	*N/A*	*N/A*	
	52	9(477)	-0.30	-0.48 to -0.11	0.0018	8.77	8.8	14.25	
	104	1(48)	-0.35	-0.92 to 0.22	*N/A*	*N/A*	*N/A*	*N/A*	

***K (N)*** denotes the number of effect sizes included in the pooled estimate (and the total number of participants contributing to that estimate); A negative value indicates that resistance training alone, or resistance training combined with aerobic training, improves arterial stiffness; ***P d*** = The p value for the pooled effect estimate.; **Power** = The statistical power of the pooled subgroup estimate.; ***P m*** = the p value for between-subgroup differences.; ***N/A*** indicates that data were not available.

Among the prespecified subgroup variables, mixed-sex samples showed a significant pooled effect (*g* = −0.25; *p* = 0.0031), and possible between-subgroup differences were observed for sex distribution (*p* = 0.001). Possible between-subgroup differences were also observed for arterial stiffness measure type (*p* = 0.009), with cdPWV showing the most favourable pooled estimate among the available strata (*g* = −0.63; *p* = 0.01), and for intervention modality (*p* = 0.0015), with RT+AT showing the most consistent significant pooled effect (*g* = −0.25; *p* = 0.002). Similar between-subgroup differences were observed for repetitions, sets, training frequency, time, and programme duration, with the more favourable pooled estimates generally appearing in studies using 6–15 repetitions, one set, three sessions per week, session durations of 30–60 min, and programmes lasting 52 weeks. These patterns may suggest potentially favourable intervention characteristics, but they should not be interpreted as robust dose-specific effects.

By contrast, although significant pooled effects were observed in the only estimable age subgroup (middle-aged adults, 45–64 years; *g* = −0.24; p = 0.0015) and the only estimable intensity subgroup (high-intensity protocols; *g* = −0.24; *p* = 0.0015), no between-subgroup comparison was possible. Accordingly, neither age nor training intensity should be interpreted as a confirmed moderator for this outcome.

**Figure 4:**
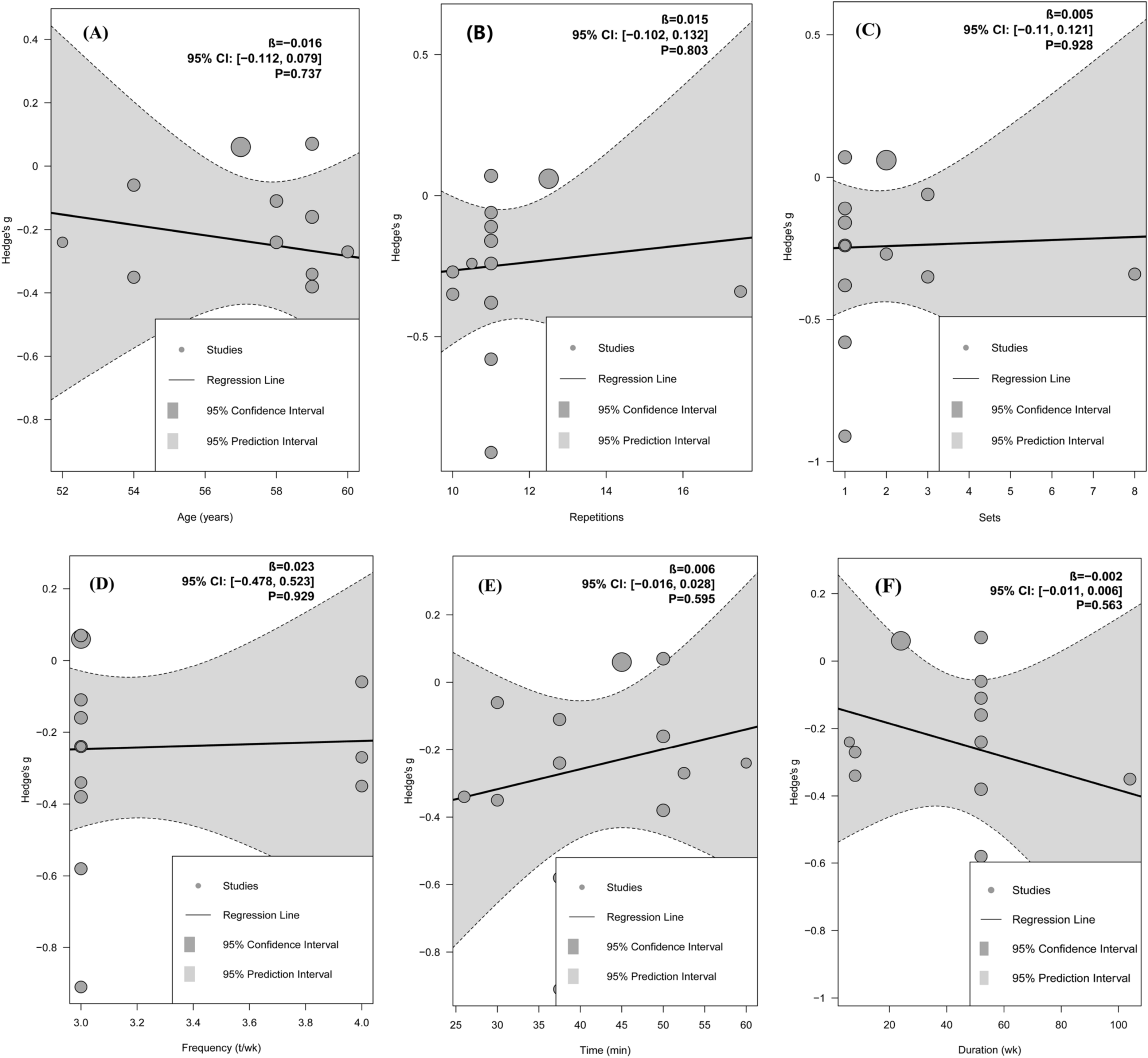
Meta-regression analysis based on meta-analysis results of arterial stiffness. **(A–F)** Meta-regression bubble plot for age, repetitions, sets, frequency, time, duration. Bubble plots for meta-regression analyses of continuous variables. Each bubble denotes an individual effect size, and bubble size is proportional to its weight in the pooled analysis. β, represents the meta-regression coefficient, indicating the estimated change in Hedge’s g for each 1-unit increase in the moderator. The black line represents the fitted regression line, the dark gray area the 95% confidence interval, and the light gray area the 95% prediction interval.

Further mixed-effects meta-regression analyses suggested that age, repetitions, number of sets, training frequency, time, and overall intervention duration may not be significantly associated with the magnitude of exercise-induced change in arterial stiffness (all *p* > 0.05; **Figure 4**).

#### 3.4.2 Effects of resistance training alone or combined with aerobic training on FMD in adults with type 2 diabetes

**Figure 5:**
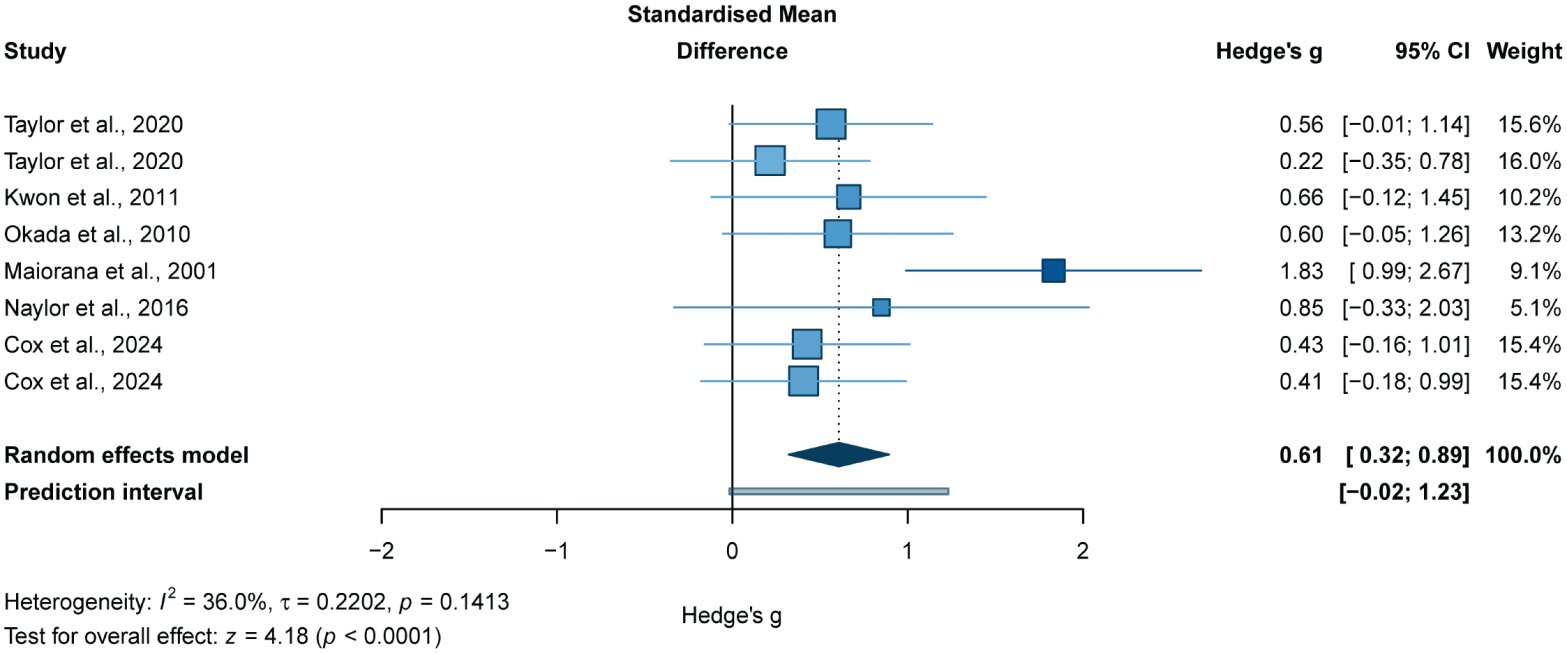
Forest plot for FMD. Forest plot of the effects of resistance training alone or resistance training combined with aerobic training on FMD in adults with T2DM, compared with non-exercise/usual-care controls; 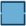, squares represent study-specific effect estimates, with square area proportional to study weight; 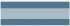, horizontal lines indicate 95% confidence intervals; 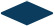, The diamond represents the pooled effect estimate, and its width indicates the 95% CI. Positive values favour exercise for FMD.

The meta-analysis of six studies (eight effect sizes; 298 participants) suggested that, compared with non-exercise controls, RT-based interventions may be associated with improved FMD in adults with T2DM (*g* = 0.61, *95% CI* 0.32 to 0.89; *p* < 0.0001; **Figure 5**).

Subgroup analyses for this outcome were performed according to participant characteristics and intervention-prescription features, with subgroup pooled effects estimated using the same random-effects model as in the primary analysis. The findings appeared to suggest possible between-subgroup differences for age, sex, intervention modality, intensity, sets, training frequency, session duration, and overall intervention duration (*p<* 0.05; **Table 3**). However, these patterns should be interpreted cautiously because several strata were informed by few studies and the subgroup analyses were exploratory in nature.

**Table 3:** Subgroup analysis of meta-analysis results of FMD.

Subgroup		K(N)	Hedges’g	95% CI	P _d_	Q	I ^2^ (%)	Power(%)	P _m_
Age									0.0001
	Young Adult: 19–24	1(13)	0.85	-0.33 to 2.03	*N/A*	*N/A*	*N/A*	*N/A*	
	Adults aged 25–44	*N/A*	*N/A*	*N/A*	*N/A*	*N/A*	*N/A*	*N/A*	
	Middle Aged: 45–64	7(285)	0.60	0.29 to 0.90	0.0001	10.73	44.1	44.29	
	Aged: ≥65	*N/A*	*N/A*	*N/A*	*N/A*	*N/A*	*N/A*	*N/A*	
Sex									0.0001
	Male	*N/A*	*N/A*	*N/A*	*N/A*	*N/A*	*N/A*	*N/A*	
	Female	1(27)	0.66	-0.12 to 1.45	*N/A*	*N/A*	*N/A*	*N/A*	
	Mixed-sex	7(271)	0.62	0.28 to 0.95	0.0003	10.89	44.9	45.46	
Intervention									0.0003
	RT	3(123)	0.44	0.09 to 0.80	0.01	1.08	0	10	
	RT+AT	5(175)	0.76	0.26 to 1.26	0.002	8.89	55	51.61	
Intensity									0.0002
	Bodyweight	2(96)	0.39	-0.02 to 0.79	0.06	0.7	0	10	
	Low intensity	1(27)	0.66	-0.12 to 1.45	*N/A*	*N/A*	*N/A*	*N/A*	
	Moderate intensity	1(46)	0.41	-0.18 to 0.99	*N/A*	*N/A*	*N/A*	*N/A*	
	High intensity	4(129)	0.88	0.25 to 1.52	0.006	7.69	61	54.37	
	To failure	*N/A*	*N/A*	*N/A*	*N/A*	*N/A*	*N/A*	*N/A*	
Repetitions									0.1
	10-25	3(119)	0.47	0.10 to 0.84	0.01	0.3	0	10	
	to failure	1(32)	1.83	0.99 to 2.67	*N/A*	*N/A*	*N/A*	*N/A*	
Sets									0.006
	3	1(27)	0.66	-0.05 to 1.26	*N/A*	*N/A*	*N/A*	*N/A*	
	8	2(92)	0.42	0.00 to 0.83	0.04	0.002	0	10	
Frequency(t/wk)									0.001
	3	4(118)	0.91	0.27 to 1.55	0.005	7.44	59.7	52.28	
	3-5	2(84)	0.49	0.06 to 0.93	0.02	0.19	0	10	
Time(min)									0.0001
	≤26	3(142)	0.40	0.07 to 0.73	0.01	0.71	0	10	
	27-59	2(73)	0.50	0.03 to 0.97	0.03	0.26	0	10	
	≥60	3(83)	1.09	0.29 to 1.88	0.0075	5.22	61.7	46.66	
Duration(wk)									0.0002
	Acute	2(96)	0.39	-0.02 to 0.79	0.06	0.7	0	10	
	8	3(124)	0.84	-0.03 to 1.72	0.05	8.75	77.2	74.92	
	12	3(78)	0.66	0.20 to 1.12	0.005	0.13	0	10	

***K (N)*** denotes the number of effect sizes included in the pooled estimate (and the total number of participants contributing to that estimate); A positive value indicates that resistance training alone, or resistance training combined with aerobic training, improves FMD; ***P d*** = The p value for the pooled effect estimate.; **Power** = The statistical power of the pooled subgroup estimate.; ***P m*** = the p value for between-subgroup differences.; ***N/A*** indicates that data were not available.

Among the prespecified subgroup variables, middle-aged adults showed a significant pooled effect (*g* = 0.60; *p* = 0.0001), and possible between-subgroup differences were observed for age (*p* = 0.0001). Possible between-subgroup differences were also observed for sex distribution (*p* = 0.0001), with mixed-sex samples showing the most favourable pooled estimate among the available strata (*g* = 0.62; *p* = 0.0003). Intervention modality was also associated with possible between-subgroup differences (*p* = 0.0003), with both RT alone (*g* = 0.44; *p* = 0.01) and RT+AT (*g* = 0.76; *p* = 0.002) showing significant pooled effects, and the latter yielding the more favourable estimate. Similar between-subgroup differences were observed for intensity, sets, training frequency, session duration, and overall intervention duration, with the more favourable pooled estimates generally appearing in studies using high-intensity protocols, eight sets, training frequencies of three sessions per week, session durations of ≥ 60 min, and programmes lasting 12 weeks. These patterns may suggest potentially favourable intervention characteristics, but they should not be interpreted as robust dose-specific effects. No significant moderating effect of repetition range was observed for FMD (*p* > 0.05).

**Figure 6:**
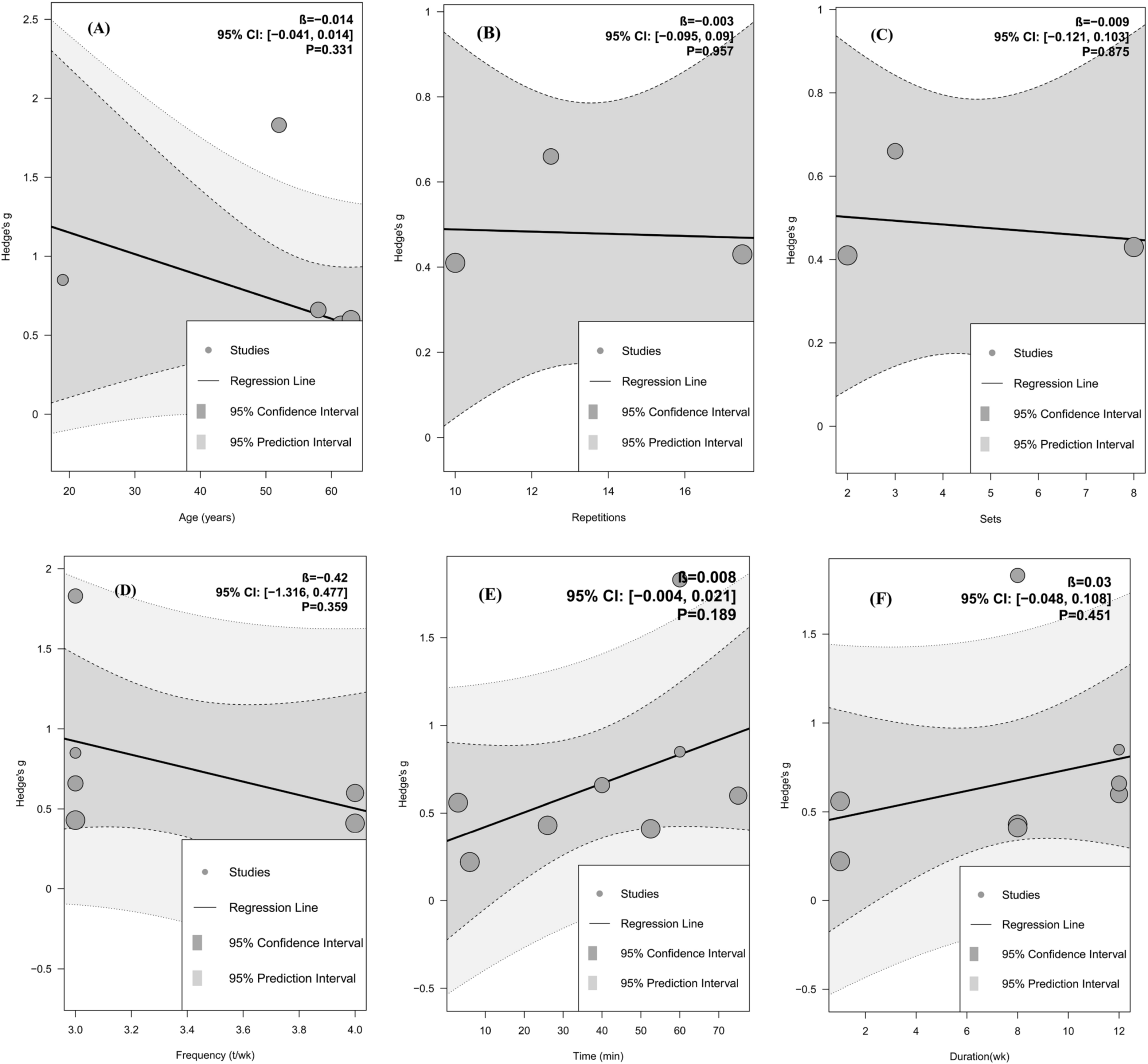
Meta-regression analysis based on meta-analysis results of FMD. **(A–F)** Meta-regression bubble plot for age, repetitions, sets, frequency, time, duration. Bubble plots for meta-regression analyses of continuous variables. Each bubble denotes an individual effect size, and bubble size is proportional to its weight in the pooled analysis. β, represents the meta-regression coefficient, indicating the estimated change in *Hedge’s g* for each 1-unit increase in the moderator. The black line represents the fitted regression line, the dark gray area the 95% confidence interval, and the light gray area the 95% prediction interval.

Further mixed-effects meta-regression analyses suggested that age, repetitions, sets, training frequency, time, and overall intervention duration may not be significantly associated with the magnitude of the exercise-related change in FMD (all *p* > 0.05; **Figure 6**).

#### 3.4.3 Effects of resistance training alone or combined with aerobic training on wave reflection indices in adults with type 2 diabetes

**Figure 7:**
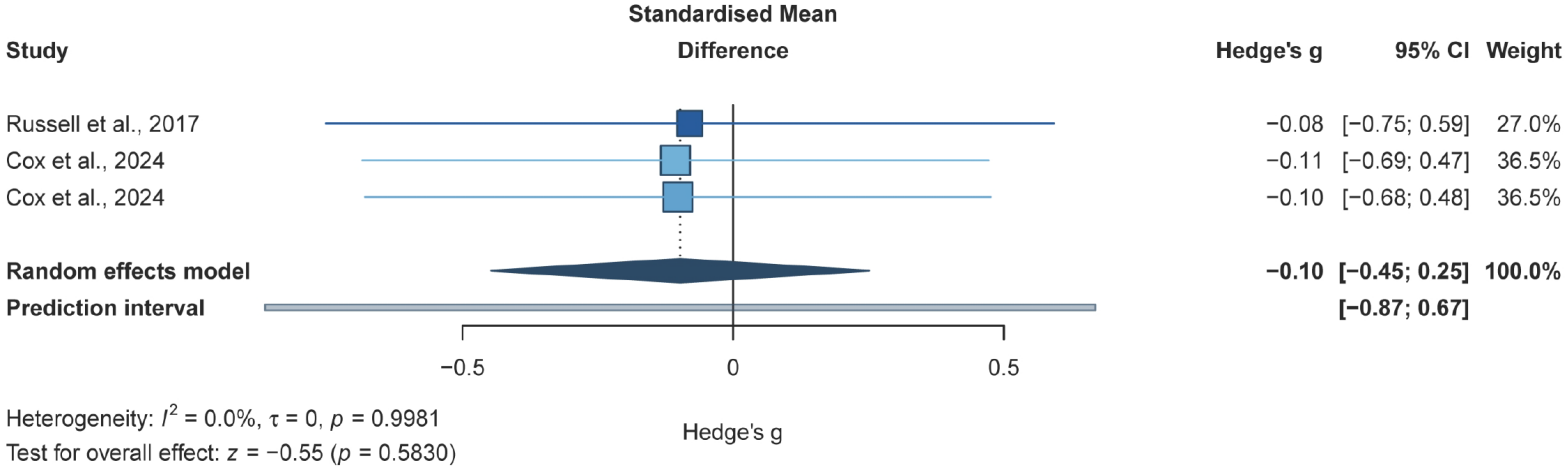
Forest plot for wave reflection indices. Forest plot of the effects of resistance training alone or resistance training combined with aerobic training on wave reflection indices in adults with T2DM, compared with non-exercise/usual-care controls; 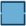, squares represent study-specific effect estimates, with square area proportional to study weight; 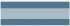, horizontal lines indicate 95% confidence intervals; 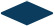, The diamond represents the pooled effect estimate, and its width indicates the 95% CI; Negative values favour exercise for wave reflection outcomes.

The meta-analysis of two studies (three effect sizes; 126 participants) suggested that, compared with non-exercise controls, RT-based interventions may not be associated with a clear improvement in wave reflection indices in adults with T2DM (*g* = −0.10, *95% CI* −0.45 to 0.25; *p* = 0.58; **Figure 7**).

#### 3.4.4 Effects of resistance training alone or combined with aerobic training on peripheral haemodynamics in adults with type 2 diabetes

**Figure 8:**
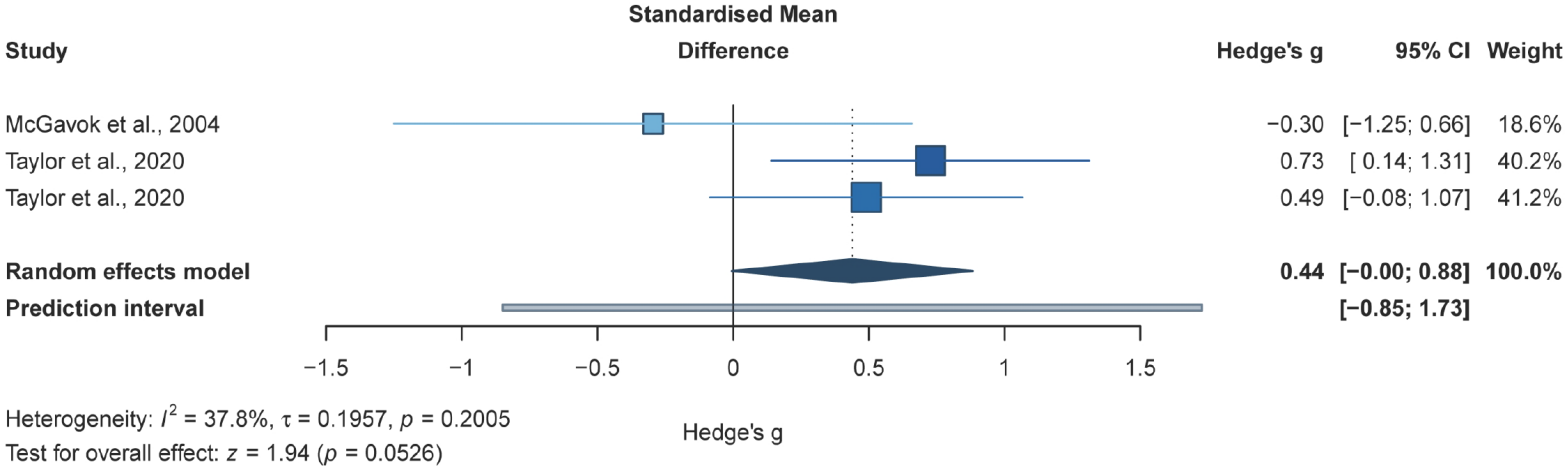
Forest plot for peripheral haemodynamics. Forest plot of the effects of resistance training alone or resistance training combined with aerobic training on peripheral haemodynamics in adults with T2DM, compared with non-exercise/usual-care controls; 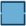, squares represent study-specific effect estimates, with square area proportional to study weight; 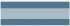, horizontal lines indicate 95% confidence intervals; 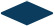, The diamond represents the pooled effect estimate, and its width indicates the 95% CI; Positive values favour exercise for peripheral haemodynamic outcomes.

The meta-analysis of two studies (three effect sizes; 114 participants) suggested that, compared with non-exercise controls, RT-based interventions may not be associated with a clear improvement in peripheral haemodynamics in adults with T2DM (*g* = 0.44, *95% CI* −0.00 to 0.88; *p* = 0.05; **Figure 8**).

### 3.5 Publication bias

**Figure 9:**
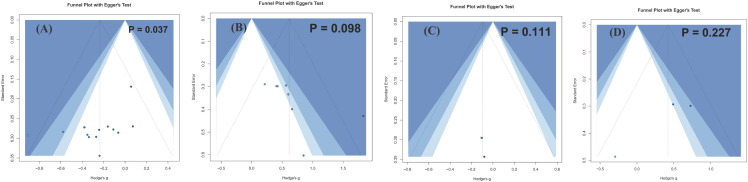
Publication bias. **(A–D)** Funnel plots for arterial stiffness, fmd, wave reflection indices, peripheral haemodynamics. Each dot represents an individual study effect size. The x-axis indicates *Hedge’s g*, and the y-axis indicates the standard error. The vertical line represents the pooled effect estimate, and the diagonal lines indicate the pseudo 95% confidence limits.

Egger’s regression test suggested possible small-study effects or publication bias for the arterial stiffness analysis (*p* < 0.05), whereas no clear evidence of publication bias was identified for the other outcomes (*p* > 0.05). The corresponding funnel plots are shown in **Figure 9**.

### 3.6 GRADE level

**Figure 10:**
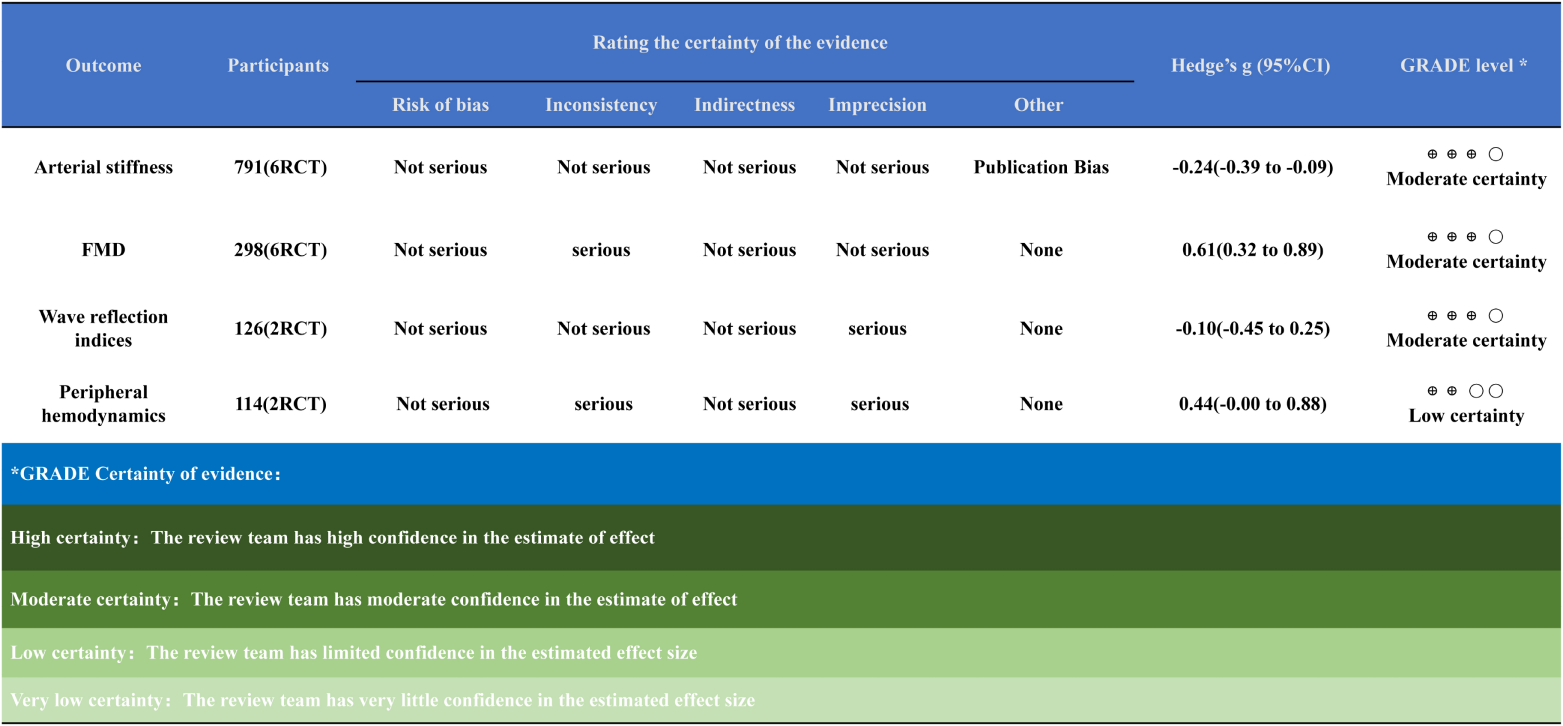
GRADE level of evidence for this study’ s findings. Randomised controlled trials start as high-certainty evidence. Certainty was downgraded by one level for each domain judged as serious and by two levels for any domain judged as very serious.

The evidence certainty (quality) ratings for the outcomes in this systematic review are presented in **Figure 10**.

## 4 Discussion

The main findings suggest that RT-based interventions confer selective vascular benefits in adults with T2DM, with the clearest support for arterial stiffness and endothelial function, whereas evidence for wave reflection indices and peripheral haemodynamics remains limited. Effects may vary according to participant and training characteristics. Importantly, these pooled findings reflect RT-based interventions overall rather than isolated RT, because most included comparisons involved combined RT+AT and modality-specific evidence remained uneven across outcomes. Moreover, although several biological pathways may plausibly contribute to these vascular adaptations, most included trials did not directly assess mechanistic variables such as nitric oxide bioavailability, insulin sensitivity, inflammatory status, oxidative stress, glycaemic control, or blood pressure trajectories. Accordingly, the mechanistic explanations discussed below should be interpreted as biologically plausible interpretations rather than findings directly established by the present meta-analysis.

### 4.1 Effects of resistance training alone or combined with aerobic training on arterial stiffness in adults with type 2 diabetes

RT-based interventions, delivered alone or in combination with aerobic training (RT+AT), were associated with a small but significant reduction in arterial stiffness in adults with T2DM, suggesting that RT-containing exercise may favourably modify an established vascular risk phenotype. Although the pooled effect was modest, this may still be clinically relevant because arterial stiffness is a recognised marker of cardiovascular risk and target-organ burden in this population (10).

Interpretation should remain cautious because arterial stiffness was assessed using related but not fully equivalent indices across studies, including PWV, cfPWV, cdPWV, crPWV, and the β-stiffness index. These measures reflect different vascular territories and may therefore not be directly comparable (56). In addition, some between-study variation may have arisen from differences in measurement approach, as devices may differ in sensing technology, waveform detection, signal processing, and the estimation of travel distance used for PWV calculation (57). It is therefore possible that part of the observed variation reflected methodological differences rather than vascular adaptation alone. Arterial stiffness measures may also be influenced by haemodynamic conditions at the time of testing. Factors such as blood pressure control, antihypertensive treatment, posture, rest duration, and recent food, caffeine, or physical activity exposure could have affected measured values independently of the intervention itself (58–60). Participant characteristics, including age, baseline vascular status, diabetes severity, adiposity, and comorbidity burden, may have further contributed to this variability. Taken together, these methodological and clinical differences may have increased between-study heterogeneity and warrant caution when interpreting the pooled standardised effect as a common estimate of clinical change across studies (58, 61).

This direction of effect is biologically plausible. However, these pathways were not directly evaluated in most included trials and therefore should not be interpreted as confirmed mediators of the pooled effect. In T2DM, arterial stiffness reflects both structural and functional vascular alterations, including extracellular matrix remodelling, vascular smooth muscle tone, and haemodynamic loading, and may be exacerbated by AGEs–RAGE-related oxidative stress and inflammation (62–64). Against this background, RT-based training may reduce the functional component of stiffness by improving haemodynamic load and peripheral vascular function, while longer interventions may also support slower structural adaptation (62, 65). When aerobic training is added, increased shear stress and nitric oxide-related signalling may provide an additional vascular-adaptive stimulus (66, 67). Improvements in insulin sensitivity and metabolic status may further reduce vascular injury associated with chronic hyperglycaemia and adiposity-related dysregulation (68, 69), although these variables were not directly assessed in most included trials. Subgroup analyses suggested more favourable patterns for combined training, higher-intensity protocols, training three times per week, sessions of 30–60 min, and longer duration, particularly 52 weeks, but these signals should be interpreted cautiously because several strata were based on few trials, dose-related variables were likely collinear, and meta-regression did not identify significant linear moderator effects (62, 67).

Taken together with previous reviews, which have highlighted limited and heterogeneous evidence for stiffness outcomes in T2DM (70, 71), the present synthesis strengthens the view that RT-containing exercise—especially RT+AT—may be useful when arterial stiffness is an explicit clinical target. This is broadly consistent with wider evidence suggesting that combined and predominantly aerobic-oriented approaches tend to show more consistent benefits than RT alone (72, 73), as well as with individual trials reporting favourable effects of structured interval or combined training on arterial stiffness in T2DM (74, 75). From a translational perspective, RT+AT may therefore be the more promising default strategy, whereas evidence for RT alone remains less certain (70, 72). Thus, when reducing arterial stiffness is specified as an explicit clinical target, the current evidence more clearly supports combined training as the default strategy, whereas RT-only prescriptions still require further confirmatory trials. Accordingly, the pooled effect for arterial stiffness should be interpreted primarily as evidence for RT-based programmes overall, with the clearest current support favouring combined RT+AT rather than isolated RT.

Practical FITT-based parameters may offer useful starting points for implementation (76), but the use of non-identical stiffness indices and the influence of concomitant therapy and delivered training dose warrant cautious interpretation. Further adequately powered trials using standardised stiffness assessment and transparent reporting of achieved training dose are needed to clarify the durability and clinical relevance of these adaptations (62, 65, 77).

### 4.2 Effects of resistance training alone or combined with aerobic training on FMD in adults with type 2 diabetes

RT-based exercise interventions, delivered either as RT alone or in combination with aerobic training (RT+AT), were associated with a statistically significant improvement in brachial artery flow-mediated dilation (FMD) in adults with T2DM, with the strongest evidentiary signal observed for combined RT+AT programmes (25, 78). This finding suggests that FMD, as an established surrogate marker of diabetic endothelial dysfunction, is responsive to structured exercise prescriptions that include a resistance-training component.

Interpretation should nevertheless remain cautious because FMD is sensitive to methodological variation. Some between-study differences may have reflected the measurement approach itself, as ultrasound systems and image-analysis methods can differ in temporal resolution, edge-detection procedures, and the identification of baseline and peak arterial diameter (79). Operator-related factors may also have contributed, given the dependence of FMD assessment on consistent probe positioning, stable image acquisition, and accurate capture of post-occlusion dilation (80). Pre-test standardisation is another likely source of variability, as fasting status, recent caffeine or physical activity exposure, time of day, rest duration, cuff position, and the analytic window used to define peak dilation may each influence the measured response independently of training effects (79, 80). Additional variability may also have arisen from background pharmacotherapy, haemodynamic conditions at the time of testing, and participant characteristics such as age, baseline endothelial status, diabetes severity, adiposity, and comorbidity burden. Taken together, these methodological and clinical differences may have contributed to between-study heterogeneity and warrant caution when interpreting the pooled standardised effect as a common estimate of clinical change across studies (81).

The potential benefit of combined RT+AT appears physiologically plausible. Accordingly, the following mechanistic interpretations should be viewed as biologically plausible rather than directly established by the present meta-analysis. FMD reflects endothelium-dependent vasodilatory responsiveness, predominantly mediated by nitric oxide-related pathways, and is sensitive to both acute haemodynamic conditions and longer-term endothelial remodelling (82). Aerobic training provides a sustained laminar shear stimulus, whereas resistance training imposes intermittent pressure-flow transients; combined training may therefore deliver complementary haemodynamic inputs that enhance endothelial adaptation (83). At the same time, variability in peak-dilation timing and non-standardised assessment protocols may contribute materially to between-study heterogeneity (82, 84). In T2DM, oxidative stress, inflammation, dysregulated endothelial signalling, and glycaemic variability may impair nitric oxide bioavailability and endothelial homeostasis, whereas exercise training may attenuate these disturbances through favourable effects on blood pressure, vascular tone, metabolic control, and inflammatory and oxidative pathways (85–88). Accordingly, the overall pattern observed in the present synthesis may be broadly consistent with a multifactorial model of endothelial injury in T2DM, although these pathways were not directly tested in most included trials.

Subgroup analyses suggested larger pooled effects for RT+AT than RT alone, as well as for higher-intensity protocols, longer session durations (≥ 60 min), and 12-week programmes. However, these findings should not be interpreted as deterministic prescription rules. Meta-regression did not identify significant linear associations between continuous moderators and FMD effect size, and the sparse evidence base, likely collinearity among prescription variables, and the possibility of non-linear or threshold effects all limit causal interpretation. These subgroup patterns are therefore better regarded as hypothesis-generating than prescriptive. Thus, for endothelial function, the present synthesis more strongly supports combined RT+AT as the dominant signal within RT-based interventions, while evidence for RT alone remains comparatively less certain.

The present synthesis adds to the literature by foregrounding FMD as a principal vascular outcome in a T2DM-specific, RT-focused framework. Its overall direction is consistent with prior evidence suggesting that exercise training can improve FMD and that combined or multi-component programmes tend to yield more consistent benefits (78, 89). However, the evidence base also included short-duration resistance-type sedentary-break interventions, which are not physiologically equivalent to conventional periodised training and may inflate between-study variability when pooled together (90, 91). This also highlights that future trials and evidence syntheses should more explicitly distinguish between evidence pertaining to acute modulation of endothelial reactivity and that reflecting chronic endothelial remodelling, and align outcome frameworks and analytic plans accordingly.

From a clinical perspective, FMD may reasonably be regarded as a training-responsive intermediate vascular outcome in T2DM, given its prognostic relevance in prospective studies (82, 92, 93). When endothelial dysfunction is prioritised as a vascular target, combined RT+AT appears to be the more promising default strategy, whereas RT alone may remain a pragmatic alternative when combined training is less feasible (94).

Interpretation should nevertheless remain cautious because the number of contributing trials was small, FMD protocols varied substantially, subgroup inference is vulnerable to ecological confounding and multiplicity, and background pharmacotherapy together with blood pressure and glycaemic trajectories were inconsistently reported (82, 84, 87, 88).

Future adequately powered head-to-head trials should compare RT versus RT+AT with explicit matching for training time and/or energy expenditure, standardise FMD assessment and reporting, and incorporate concurrent mechanistic measures such as nitric oxide-related indices, glycaemic control, blood pressure, and inflammatory and oxidative stress biomarkers to distinguish transient functional changes from more durable endothelial remodelling (87).

### 4.3 Effects of resistance training alone or combined with aerobic training on wave reflection indices in adults with type 2 diabetes

This analysis examined the effects of RT-based exercise interventions, delivered either as RT+AT, on wave reflection indices in adults with T2DM. In the currently available randomised evidence, RT-based interventions did not produce a statistically significant change relative to non-exercise/usual-care controls (two trials; three effect sizes; n = 126; *g* = −0.10, *95% CI* −0.45 to 0.25; *p* = 0.58), indicating that the current evidence is insufficient to establish a consistent effect on reflected-wave load.

Wave reflection remains clinically relevant because, together with arterial stiffness and peripheral vascular tone, it contributes to central haemodynamics and left ventricular afterload (95). A neutral pooled effect is physiologically plausible even when other vascular domains improve, because indices such as augmentation index (AIx) are integrated system-level signals rather than readouts of a single vascular component. AIx is influenced not only by pulse wave velocity and return time of reflected waves, but also by heart rate, mean arterial pressure, body size, left ventricular ejection dynamics, myocardial contractility, and ventricular–arterial coupling (96–99). In T2DM, this complexity may be greater still, as observational data suggest that arterial stiffness and pulse pressure can be elevated without a commensurate increase in AIx, implying that AIx may be an unreliable surrogate for stiffness in this population (100–102).

From an evidence-synthesis perspective, the pooled estimate for wave reflection indices (*g* = −0.10, *95% CI* −0.45 to 0.25; *p* = 0.58) may be more appropriately interpreted as reflecting imprecision, rather than as indicating a clear absence of effect. Only two trials contributed data, the confidence interval was wide, and methodological heterogeneity in AIx assessment was likely substantial. Some of this variation may have arisen from the measurement system itself, because AIx can differ according to device type, calibration procedure, transfer function, and signal-processing algorithm, and these approaches are not necessarily interchangeable (103). Operator-related factors may also have contributed, particularly where waveform acquisition depends on stable applanation, signal quality, and appropriate acceptance of technically adequate recordings (104). In addition, pre-test standardisation is likely to be important, as AIx is sensitive to haemodynamic conditions at the time of testing; factors such as heart rate, blood pressure, posture, rest duration, time of day, and recent food, caffeine, or physical activity exposure may all influence the measured value independently of training adaptation (105). Background pharmacotherapy may have introduced further heterogeneity, because antihypertensive and other vasoactive agents can alter central pressure and wave reflection characteristics irrespective of exercise effects (106). Participant characteristics, including age, body size, baseline arterial stiffness, diabetes severity, and comorbidity burden, may also have influenced both absolute AIx values and the extent to which wave reflection responded to training, given that age, heart rate, and body size are established determinants of augmentation index (107, 108). Taken together, these methodological and clinical differences may have increased between-study heterogeneity, reduced comparability across trials, and lowered confidence in the pooled estimate. Accordingly, the present result is better interpreted as inconclusive rather than as evidence that RT-based exercise has no effect on wave reflection indices.

The present review adds value by treating wave reflection as a distinct vascular domain rather than inferring it indirectly from PWV or FMD. This is important because improvements in arterial stiffness or endothelial function do not necessarily imply parallel reductions in central wave-related haemodynamic load. Consistent with broader exercise-physiology evidence, wave reflection indices appear better suited as mechanistic or secondary endpoints than as primary adjudication criteria in small RCTs (109). Accordingly, until the evidence base expands and measurement protocols become more harmonised, AIx and related indices should be interpreted cautiously and preferably used as prespecified secondary outcomes. Future studies should prioritise adequately powered, time- and/or energy-matched comparisons of RT versus RT+AT, standardised AIx measurement and reporting, explicit documentation of medication stability and haemodynamic trajectories, and, where feasible, concurrent reporting of wave-separation metrics to reduce interpretive ambiguity (99, 110).

### 4.4 Effects of resistance training alone or combined with aerobic training on peripheral haemodynamics in adults with type 2 diabetes

This analysis examined whether RT-based interventions may influence peripheral haemodynamics in adults with T2DM. In the available randomised evidence, no clear improvement was observed relative to non-exercise/usual-care controls, and the pooled estimate was imprecise, remaining compatible with effects ranging from trivial to potentially clinically meaningful benefit (*g* = 0.44, *95% CI* −0.00 to 0.88; *p* = 0.05). The current evidence therefore appears insufficient to establish whether RT-based training exerts a consistent effect on peripheral haemodynamics.

A key difficulty in interpretation is that peripheral haemodynamics in this review did not represent a single homogeneous construct. The pooled estimate combined endpoints such as blood flow (BF) and systemic vascular resistance (SVR), which differ fundamentally in their physiological determinants and measurement pathways. BF is highly state-dependent and sensitive to posture, ambient temperature, sympathetic-local vasomotor control, and the timing and standardisation of assessment, including recent food intake, caffeine exposure, and medication use (111). By contrast, SVR is derived from arterial pressure and cardiac output and is therefore influenced by the underlying haemodynamic model and device-specific algorithms, with its precision varying according to how cardiac output is estimated (112). Even under controlled conditions, peripheral Doppler-based assessments show non-trivial operator- and protocol-dependent variability (113). Methodological variation may have arisen not only from the use of different endpoint constructs, but also from differences in the measurement systems themselves. Different devices and analytic approaches may not be fully interchangeable and, for BF in particular, reproducibility may also be affected by operator-dependent factors such as probe positioning, insonation angle, signal acquisition quality, and the acceptance of technically adequate recordings (114, 115). Pre-test standardisation may represent an additional source of variation, because peripheral haemodynamic measures are sensitive to posture, rest duration, environmental conditions, and recent exposure to food, caffeine, medication, or physical activity (116, 117). Background pharmacotherapy and prevailing haemodynamic conditions may also have introduced heterogeneity, as blood-pressure trajectories and vasoactive treatment can influence BF and SVR independently of exercise effects (106). Participant characteristics, including age, adiposity, baseline microvascular status, diabetes severity, and comorbidity burden, may have further contributed to variation in both absolute values and training responsiveness, given the established links between obesity, insulin resistance, and skeletal muscle microvascular dysfunction (118). Collectively, these methodological and clinical differences may have increased between-study heterogeneity, reduced comparability across trials, and lowered confidence in the pooled estimate. Pooling endpoints that reflect distinct constructs using standardised differences is therefore likely to dilute any true intervention effect through construct heterogeneity and measurement noise.

From a biological perspective, the absence of a clear pooled improvement is not necessarily inconsistent with vascular benefit; it may instead reflect domain-specific responsiveness and limited sensitivity of the selected endpoints. Accordingly, the mechanistic explanations that follow are proposed as biologically plausible rather than as findings directly established by the present meta-analysis. In adults with T2DM, exercise training may improve microvascular vasomotor signalling, perfusion distribution, and, over longer time scales, structural microvascular phenotypes (119, 120). However, such adaptations are likely to be detected more readily using reactive or challenge-based microvascular outcomes than resting BF or model-derived SVR. Consistent with this view, short-term RT has been shown to improve skeletal muscle microvascular blood flow responses to an oral glucose challenge, with such changes linked to better glycaemic control (54). Systematic review evidence on lower-limb perfusion in diabetes also suggests a possible benefit of physical activity, although substantial heterogeneity in intervention design, measurement methodology, and sample size continues to limit stable inference, particularly for resting peripheral haemodynamic outcomes (121). Similarly, null findings for foot skin microvascular reactivity in supervised exercise trials may reflect limited statistical power and/or meaningful differences in delivered dose and participant phenotype rather than true absence of effect (122). Collectively, these findings suggest that the peripheral haemodynamics domain requires more granular outcome selection, particularly a clearer distinction between resting and reactive endpoints, together with stronger measurement harmonisation.

Methodological constraints are therefore likely to dominate the current evidence base. Only two trials contributed peripheral haemodynamic data, and they differed materially in both intervention structure and outcome operationalisation. In this context, the pooled estimate for peripheral haemodynamics (*g* = 0.44, *95% CI* −0.00 to 0.88; *p* = 0.05) may be more appropriately interpreted as inconclusive, rather than as indicating a clear effect of RT-based exercise on peripheral haemodynamics. Peripheral haemodynamic measures are also highly sensitive to device modality, analytic model, posture, environmental conditions, and timing relative to food intake, caffeine, and medication use, all of which inflate variance and reduce statistical power in small trials. Moreover, background pharmacotherapy and blood-pressure trajectories are not peripheral considerations for BF and SVR, but central determinants that may confound or mediate the observed response. In this context, the current findings should be interpreted as refining expectations rather than negating the established benefits of exercise in T2DM. The broader literature consistently supports exercise training for improving glycaemic control and cardiometabolic risk factors (15, 16, 31), while vascular-focused syntheses suggest that endothelial outcomes are more consistently responsive than integrated central or peripheral haemodynamic indices (23, 25, 35). This asymmetry is biologically plausible, as endothelial reactivity may respond more readily to repeated shear-related stimuli, whereas resting BF and SVR are more constrained by microvascular rarefaction, autonomic regulation, and pharmacologically controlled blood pressure (17). By treating peripheral haemodynamics as a distinct outcome domain rather than inferring it indirectly from FMD or PWV, the present synthesis reduces overgeneralisation and highlights an under-characterised gap in RT-centred trials in adults with T2DM.

Accordingly, the most defensible next step is not broader extrapolation from a borderline pooled estimate, but adequately powered trials with standardised assessment protocols to test whether sustained, clearly quantified RT+AT can elicit detectable changes in resistance-vessel tone and peripheral perfusion alongside improvements in more sensitive vascular phenotypes such as FMD and arterial stiffness.

### 4.5 Clinical implications

From a clinical and translational perspective, RT-based exercise may contribute to vascular management in adults with T2DM, although the current evidence more consistently favours combined RT+AT than isolated RT. Across subgroup analyses, the most consistent directional patterns for the two principal benefit outcomes—arterial stiffness and endothelial function—favoured combined training, higher-intensity protocols, and, in several comparisons, training frequencies of approximately three sessions per week. However, these patterns should be interpreted as exploratory rather than prescriptive, because exercise-prescription variables were not reported consistently and several strata were informed by only a small number of studies. Accordingly, these findings may offer provisional guidance for programme design, but they do not constitute definitive evidence for an optimal prescription, particularly in light of between-study heterogeneity and the absence of significant linear associations in meta-regression analyses.

The current evidence also suggests that prescription priorities may vary according to the vascular outcome targeted. For endothelial function, more favourable patterns were generally observed with longer session durations and programmes lasting at least 12 weeks, whereas for arterial stiffness, benefits tended to cluster around session durations of 30–60 min and longer intervention exposure. However, these patterns should be interpreted as exploratory rather than as definitive prescription thresholds. Taken together, the findings suggest potential clinical value for RT-containing programmes, particularly combined RT+AT, while also indicating that exercise prescription may need to be tailored to the vascular phenotype of interest rather than assumed to be uniform across outcomes. More definitive recommendations will require adequately powered, dose-matched trials with harmonised training protocols and standardised vascular assessments.

## 5 Limitations

This review has several limitations. Evidence for wave reflection and peripheral haemodynamics was based on only two studies, resulting in imprecise estimates. Although more studies were available for arterial stiffness and flow-mediated dilation, several subgroup analyses still relied on few effect sizes. Reporting of exercise-prescription variables was also incomplete across studies: while modality, frequency, session duration, and programme duration were usually available, repetitions, sets, supervision, progression, and achieved dose/adherence were less consistently reported, limiting the robustness of moderator analyses and the strength of practical prescription inferences. Heterogeneity in intervention characteristics and comparator conditions may also have influenced the pooled results despite the use of random-effects models and subgroup analyses. In addition, incomplete reporting of randomisation, allocation concealment, blinding, and selective reporting in several trials increases concerns about risk of bias. Finally, study-level moderator analyses were constrained by collinearity and limited statistical power and should therefore be interpreted as exploratory.

## 6 Future directions

Future studies should prioritise under-represented outcomes, particularly wave reflection and peripheral haemodynamics. More rigorous and standardised trials are needed to clarify the effects of different exercise modes and prescriptions, improve measurement consistency, and integrate vascular outcomes with mechanistic measures such as nitric oxide-related indices, insulin sensitivity, glycaemic control, blood pressure, and inflammatory and oxidative stress biomarkers. Standardised and transparent reporting of exercise-prescription variables, including supervision, progression, achieved dose, and adherence, will also be essential to improve the interpretability of dose-related moderator analyses. Greater inclusion of women and older adults, together with longer follow-up, is needed to enhance generalisability and clinical relevance.

## 7 Conclusion

This review suggests that RT-based exercise, particularly combined RT+AT, may benefit vascular management in adults with T2DM. Vascular responses appear selective across domains, with the clearest potential benefits observed for arterial stiffness and endothelial function. However, because the pooled estimates reflect RT-based programmes overall and evidence for RT alone was more limited, conclusions regarding isolated RT should remain cautious. Further well-designed trials are needed before more definitive prescription recommendations can be made.

## Data Availability

The raw data supporting the conclusions of this article will be made available by the authors without reservation.

## Glossary

T2DM: type 2 diabetes mellitus
CVD: cardiovascular disease
IDF: International Diabetes Federation
RT: resistance training
AT: aerobic training
RT+AT: resistance training plus aerobic training
FMD: flow-mediated dilation
PWV: pulse wave velocity
cfPWV: carotid-femoral pulse wave velocity
cdPWV: carotid to distal posterior tibial artery pulse wave velocity
crPWV: carotid to radial artery pulse wave velocity
AIx: augmentation index
BF: blood flow
SVR: systemic vascular resistance
PVR: peripheral vascular resistance
PRISMA: Preferred Reporting Items for Systematic Reviews and Meta-Analyses
PROSPERO: International Prospective Register of Systematic Reviews
CNKI: China National Knowledge Infrastructure
VIP: China Science and Technology Journal Database
CBM: Chinese Biomedical Literature Database
MeSH: Medical Subject Headings
PICOS: Population, Intervention, Comparator, Outcomes, and Study design
CI/CIs: confidence interval/confidence intervals
SD: standard deviation
SE: standard error
MD/MDs: mean difference/mean differences
SMD/SMDs: standardised mean difference/standardised mean differences
RCT/RCTs: randomised controlled trial/randomised controlled trials
RevMan: Review Manager
REML: restricted maximum likelihood
GRADE: Grading of Recommendations Assessment, Development and Evaluation
SRA: simple resistance activities
SRA3: 3-min simple resistance activities
SRA6: 6-min simple resistance activities
HRR: heart rate reserve
1RM: one-repetition maximum
12RM: 12-repetition maximum
HRmax: maximal heart rate
HRpeak: peak heart rate
MVC: maximal voluntary contraction
HIIT: high-intensity interval training
RPE: rating of perceived exertion
VO_2_max: maximal oxygen uptake
VO_2_peak: peak oxygen uptake
AEP: accredited exercise physiologist
NO: nitric oxide
AGE/AGEs: advanced glycation end product/advanced glycation end products
RAGE: receptor for advanced glycation end products
FITT: frequency, intensity, time, and type
MCT: moderate continuous training
MICT/C-MICT: moderate-intensity continuous training/combined moderate-intensity continuous training
AIx@75: augmentation index normalized to a heart rate of 75 beats/min
Pb/Pf: backward-to-forward pressure ratio
M/F: male/female
NR: not reported
N/A: not available
K(N): number of effect sizes (K) and total participants (N)
P d: p value for pooled effect estimate
P m: p value for between-subgroup differences
