# Supplementary material for: Does resistance training alone or in combination with aerobic training improve vascular function indices in adults with type 2 diabetes? A systematic review and meta-analysis of randomized controlled trials
Source: Front Endocrinol (Lausanne). 2026 May 15;17:1824213. doi: 10.3389/fendo.2026.1824213 (PMC13218868; doi:10.3389/fendo.2026.1824213)

| X：Duration(wk) | Y：（effect size）Hedge's g | Weight（%） |
| --- | --- | --- |
| 24 | 0.06 | 15.7 |
| 104 | -0.35 | 6.3 |
| 52 | -0.06 | 6.5 |
| 6 | -0.24 | 4.6 |
| 8 | -0.34 | 6.0 |
| 8 | -0.27 | 6.1 |
| 52 | 0.07 | 7.2 |
| 52 | -0.11 | 6.8 |
| 52 | -0.38 | 7.1 |
| 52 | -0.91 | 6.2 |
| 52 | -0.16 | 7.2 |
| 52 | -0.58 | 6.6 |
| 52 | -0.38 | 7.1 |
| 52 | -0.24 | 6.8 |

# 加载必要的包

library(metafor)

# 创建数据框（已替换为 Duration.docx 中的数据）

df <- data.frame(

Duration = c(24, 104, 52, 6, 8, 8, 52, 52, 52, 52, 52, 52, 52, 52),

g = c(0.06, -0.35, -0.06, -0.24, -0.34, -0.27, 0.07, -0.11, -0.38, -0.91, -0.16, -0.58, -0.38, -0.24),

Weight = c(15.7, 6.3, 6.5, 4.6, 6.0, 6.1, 7.2, 6.8, 7.1, 6.2, 7.2, 6.6, 7.1, 6.8)

)

# 计算方差（按你原代码逻辑：权重为 1/vi）

df$vi <- 1 / df$Weight

# 执行 Meta 回归分析（混合效应模型）

res <- rma(yi = g, vi = vi, mods = ~ Duration, data = df)

# 提取统计结果（稳健写法：从 summary(res) 的系数表取数值）

tab <- coef(summary(res)) # estimate, se, zval, pval, ci.lb, ci.ub

beta <- round(tab[2, "estimate"], 3)

ci_lb <- round(tab[2, "ci.lb"], 3)

ci_ub <- round(tab[2, "ci.ub"], 3)

p_value <- ifelse(tab[2, "pval"] < 0.001, "< 0.001", round(tab[2, "pval"], 3))

# 绘制气泡图

regplot(

res,

mod = "Duration",

pi = TRUE,

pred = TRUE,

xlab = "Duration (wk)",

ylab = "Hedge's g",

psize = sqrt(df$Weight),

col = "black",

ci.col = "darkgray",

pi.col = "lightgray",

las = 1

)

# 添加统计结果文本

text(

x = max(df$Duration) - 0.2*(max(df$Duration) - min(df$Duration)),

y = max(df$g) - 0.1*(max(df$g) - min(df$g)),

labels = paste0(

"β=", beta, "\n",

"95% CI: [", ci_lb, ", ", ci_ub, "]\n",

"P=", p_value

),

pos = 2,

cex = 0.9,

col = "black",

font = 2

)

# 添加紧凑图例（注意：去掉了你原代码里 bg 后面的多余逗号）

legend(

"bottomright",

legend = c("Studies", "Regression Line", "95% Confidence Interval", "95% Prediction Interval"),

pch = c(19, NA, NA, NA),

lty = c(NA, 1, NA, NA),

fill = c(NA, NA, "darkgray", "lightgray"),

border = c(NA, NA, "darkgray", "lightgray"),

col = c("gray60", "black", NA, NA),

pt.cex = 1.0,

cex = 0.62,

x.intersp = 0.75,

y.intersp = 0.75,

bg = "white"

)


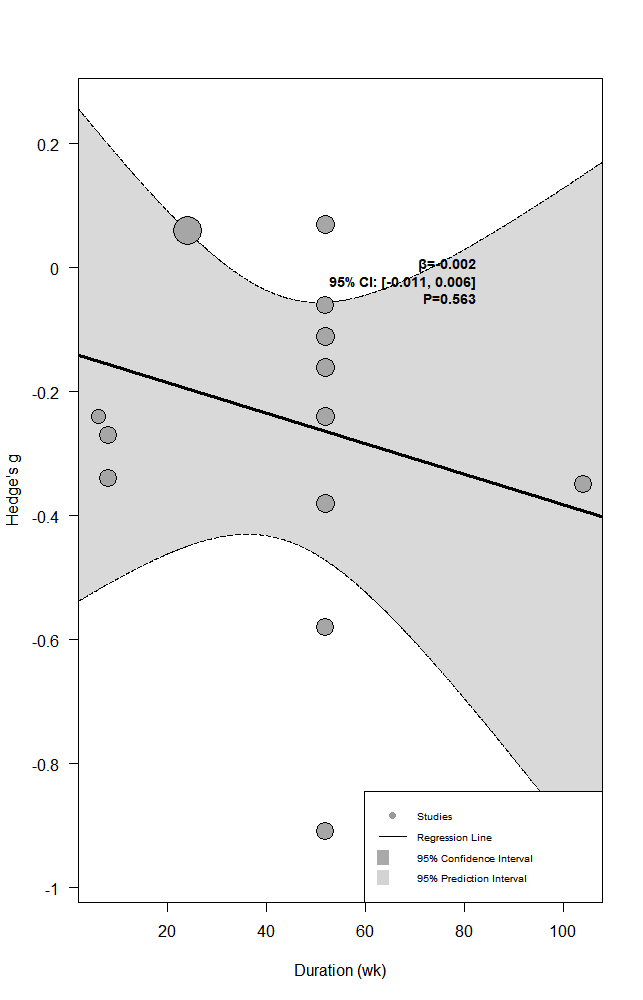

Supplement: Supplementary file 1 [file DataSheet1.zip › Supplementary File/Arterial stiffness/Meta-regression analysis/Duration.docx]
