# Supplementary material for: Does resistance training alone or in combination with aerobic training improve vascular function indices in adults with type 2 diabetes? A systematic review and meta-analysis of randomized controlled trials
Source: Front Endocrinol (Lausanne). 2026 May 15;17:1824213. doi: 10.3389/fendo.2026.1824213 (PMC13218868; doi:10.3389/fendo.2026.1824213)

| Study | Experiment | | | Control | | |
| --- | --- | --- | --- | --- | --- | --- |
|  | Total | MEAN | SD | Total | MEAN | SD |
| Dobrosielski et al., 2012 | 70 | 929.6 | 332.1 | 70 | 910.1 | 360.4 |
| Loimaala et al., 2009 | 24 | 14.7 | 1.96 | 24 | 15.4 | 1.96 |
| Loimaala et al., 2003 | 24 | 14.8 | 2.3 | 25 | 15.0 | 3.7 |
| Russell et al., 2017 | 17 | 7.6 | 0.82 | 17 | 7.8 | 0.82 |
| Cox et al., 2024 | 23 | 9.1 | 1.3 | 23 | 9.6 | 1.6 |
| Cox et al., 2024 | 23 | 9.2 | 1.3 | 23 | 9.6 | 1.6 |
| Magalhães et al., 2019 | 28 | 14.3 | 3.9 | 27 | 14.0 | 4.3 |
| Magalhães et al., 2019 | 25 | 13.5 | 4.7 | 27 | 14.0 | 4.3 |
| Magalhães et al., 2019 | 28 | 9.7 | 1.4 | 27 | 10.3 | 1.7 |
| Magalhães et al., 2019 | 25 | 8.6 | 2.0 | 27 | 10.3 | 1.7 |
| Magalhães et al., 2019 | 28 | 9.0 | 2.1 | 27 | 9.3 | 1.5 |
| Magalhães et al., 2019 | 25 | 8.3 | 1.9 | 27 | 9.3 | 1.5 |
| Magalhães et al., 2019 | 28 | 12.3 | 1.9 | 27 | 14.2 | 6.8 |
| Magalhães et al., 2019 | 25 | 12.6 | 6.2 | 27 | 14.2 | 6.8 |

# ================================

# 完整代码：森林图 + Egger + 漏斗图

# 配色：渐变蓝色系·深色增强版（森林图方块/研究名随TE渐变；菱形/线深蓝；漏斗图气泡同渐变蓝；漏斗背景等高线同蓝系）

# ================================

# 加载所需包

library(meta)

library(grid)

# 创建研究名称向量（同一文献多条记录用 ^1^,^2^... 区分）

study <- c(

"Dobrosielski et al., 2012",

"Loimaala et al., 2009",

"Loimaala et al., 2003",

"Russell et al., 2017",

"Cox et al., 2024^1^",

"Cox et al., 2024^2^",

"Magalhães et al., 2019^1^",

"Magalhães et al., 2019^2^",

"Magalhães et al., 2019^3^",

"Magalhães et al., 2019^4^",

"Magalhães et al., 2019^5^",

"Magalhães et al., 2019^6^",

"Magalhães et al., 2019^7^",

"Magalhães et al., 2019^8^"

)

# 实验组和对照组数据（来自文档表格）

n1 <- c(70, 24, 24, 17, 23, 23, 28, 25, 28, 25, 28, 25, 28, 25)

m1 <- c(929.6, 14.7, 14.8, 7.6, 9.1, 9.2, 14.3, 13.5, 9.7, 8.6, 9.0, 8.3, 12.3, 12.6)

sd1 <- c(332.1, 1.96, 2.3, 0.82, 1.3, 1.3, 3.9, 4.7, 1.4, 2.0, 2.1, 1.9, 1.9, 6.2)

n2 <- c(70, 24, 25, 17, 23, 23, 27, 27, 27, 27, 27, 27, 27, 27)

m2 <- c(910.1, 15.4, 15.0, 7.8, 9.6, 9.6, 14.0, 14.0, 10.3, 10.3, 9.3, 9.3, 14.2, 14.2)

sd2 <- c(360.4, 1.96, 3.7, 0.82, 1.6, 1.6, 4.3, 4.3, 1.7, 1.7, 1.5, 1.5, 6.8, 6.8)

# Meta分析（Hedge's g）

meta_result <- metacont(

n1, m1, sd1,

n2, m2, sd2,

studlab = study,

sm = "SMD",

method.smd = "Hedges",

comb.fixed = FALSE,

comb.random = TRUE,

method.tau = "DL",

method.tau.ci = "J",

hakn = FALSE

)

# ================================

# 渐变蓝色系·深色增强版 配色函数

# ================================

pal_fn <- grDevices::colorRampPalette(c("#6BAED6", "#3182BD", "#08519C"))

pal <- pal_fn(200)

col_line <- "#0B3C5D"

map_to_col <- function(x, pal, rng = NULL) {

if (is.null(rng)) rng <- range(x, na.rm = TRUE)

if (!is.finite(diff(rng)) || diff(rng) == 0) return(rep(pal[length(pal)], length(x)))

idx <- floor((x - rng[1]) / diff(rng) * (length(pal) - 1)) + 1

pal[pmax(1, pmin(length(pal), idx))]

}

# 森林图方块/研究名的渐变色（按TE映射）

te_rng <- range(meta_result$TE, na.rm = TRUE)

col_sq_vec <- map_to_col(meta_result$TE, pal, rng = te_rng)

# 漏斗图气泡渐变色（同样按TE映射）

col_pt_vec <- col_sq_vec

# 漏斗图等高线区域（半透明蓝渐变）

col_contour_vec <- grDevices::adjustcolor(c("#6BAED6", "#3182BD", "#08519C"), alpha.f = 0.35)

# ================================

# 1) 森林图（隐藏原始数据；渐变蓝增强）

# ================================

forest(

meta_result,

sortvar = 1:length(study),

xlab = "Hedge's g (95% CI)",

leftcols = c("studlab", "effect", "ci"),

leftlabs = c("Study", "g", "95% CI"),

rightcols = FALSE,

col.square = col_sq_vec,

col.study = col_sq_vec,

col.square.lines = col_line,

col.diamond = col_line,

col.diamond.lines= col_line,

print.tau2 = TRUE,

print.tau2.ci = TRUE,

print.tau = TRUE

)

# ================================

# 2) Egger's 检验

# ================================

egger_test <- metabias(meta_result, method = "Egger", k.min = 3)

p_value <- ifelse(

egger_test$p.value < 0.001,

"P < 0.001",

paste0("P = ", round(egger_test$p.value, 3))

)

# ================================

# 3) 漏斗图（气泡也用渐变蓝；等高线区域用蓝系）

# ================================

funnel(

meta_result,

studlab = FALSE,

contour = c(0.9, 0.95, 0.99),

col.contour = col_contour_vec,

pch = 21, # ✅ 可填充圆点

bg = col_pt_vec, # ✅ 填充渐变色

col = col_line, # ✅ 边框深蓝

xlab = "Hedge's g",

main = "Funnel Plot with Egger's Test"

)

# 添加P值标注（左上角）

text(

x = min(meta_result$TE, na.rm = TRUE) + 0.2,

y = max(meta_result$seTE, na.rm = TRUE) * 0.95,

labels = p_value,

pos = 4,

cex = 1.1,

col = "black",

font = 2

)

# 控制台输出检验结果

cat(

"Egger's Linear Regression Test:\n",

"t =", round(egger_test$statistic, 3),

"| df =", egger_test$df,

"|", p_value

)


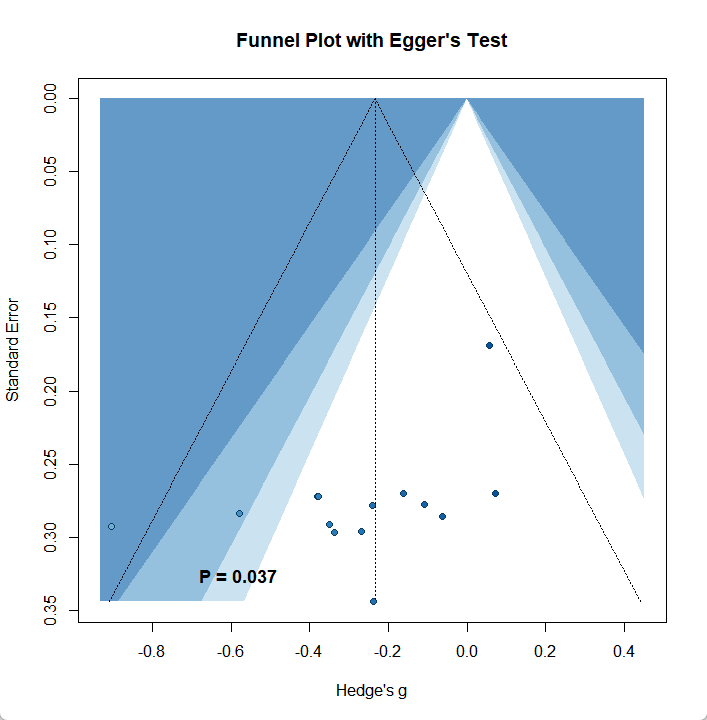

Supplement: Supplementary file 1 [file DataSheet1.zip › Supplementary File/Arterial stiffness/Publication Bias/Publication Bias.docx]
