# Supplementary material for: Does resistance training alone or in combination with aerobic training improve vascular function indices in adults with type 2 diabetes? A systematic review and meta-analysis of randomized controlled trials
Source: Front Endocrinol (Lausanne). 2026 May 15;17:1824213. doi: 10.3389/fendo.2026.1824213 (PMC13218868; doi:10.3389/fendo.2026.1824213)
Supplement: Supplementary file 1 [file DataSheet1.zip › Supplementary File/Arterial stiffness/Subgroup analysis/Age/Middle Aged 45–64.docx]

| Study | Experiment | | | Control | | |
| --- | --- | --- | --- | --- | --- | --- |
|  | Total | MEAN | SD | Total | MEAN | SD |
| Dobrosielski et al., 2012 | 70 | 929.6 | 332.1 | 70 | 910.1 | 360.4 |
| Loimaala et al., 2009 | 24 | 14.7 | 1.96 | 24 | 15.4 | 1.96 |
| Loimaala et al., 2003 | 24 | 14.8 | 2.3 | 25 | 15.0 | 3.7 |
| Russell et al., 2017 | 17 | 7.6 | 0.82 | 17 | 7.8 | 0.82 |
| Cox et al., 2024 | 23 | 9.1 | 1.3 | 23 | 9.6 | 1.6 |
| Cox et al., 2024 | 23 | 9.2 | 1.3 | 23 | 9.6 | 1.6 |
| Magalhães et al., 2019 | 28 | 14.3 | 3.9 | 27 | 14.0 | 4.3 |
| Magalhães et al., 2019 | 25 | 13.5 | 4.7 | 27 | 14.0 | 4.3 |
| Magalhães et al., 2019 | 28 | 9.7 | 1.4 | 27 | 10.3 | 1.7 |
| Magalhães et al., 2019 | 25 | 8.6 | 2.0 | 27 | 10.3 | 1.7 |
| Magalhães et al., 2019 | 28 | 9.0 | 2.1 | 27 | 9.3 | 1.5 |
| Magalhães et al., 2019 | 25 | 8.3 | 1.9 | 27 | 9.3 | 1.5 |
| Magalhães et al., 2019 | 28 | 12.3 | 1.9 | 27 | 14.2 | 6.8 |
| Magalhães et al., 2019 | 25 | 12.6 | 6.2 | 27 | 14.2 | 6.8 |

## ================================

## 0. 环境准备

## ================================

library(meta)

## ================================

## 1. 构建数据（已替换为 数据表.docx 中的数据）

## ================================

data <- data.frame(

Study = c(

"Dobrosielski et al., 2012",

"Loimaala et al., 2009",

"Loimaala et al., 2003",

"Russell et al., 2017",

"Cox et al., 2024",

"Cox et al., 2024",

"Magalhães et al., 2019",

"Magalhães et al., 2019",

"Magalhães et al., 2019",

"Magalhães et al., 2019",

"Magalhães et al., 2019",

"Magalhães et al., 2019",

"Magalhães et al., 2019",

"Magalhães et al., 2019"

),

n_e = c(70, 24, 24, 17, 23, 23, 28, 25, 28, 25, 28, 25, 28, 25),

mean_e = c(929.6, 14.7, 14.8, 7.6, 9.1, 9.2, 14.3, 13.5, 9.7, 8.6, 9.0, 8.3, 12.3, 12.6),

sd_e = c(332.1, 1.96, 2.3, 0.82, 1.3, 1.3, 3.9, 4.7, 1.4, 2.0, 2.1, 1.9, 1.9, 6.2),

n_c = c(70, 24, 25, 17, 23, 23, 27, 27, 27, 27, 27, 27, 27, 27),

mean_c = c(910.1, 15.4, 15.0, 7.8, 9.6, 9.6, 14.0, 14.0, 10.3, 10.3, 9.3, 9.3, 14.2, 14.2),

sd_c = c(360.4, 1.96, 3.7, 0.82, 1.6, 1.6, 4.3, 4.3, 1.7, 1.7, 1.5, 1.5, 6.8, 6.8)

)

## ================================

## 2. Meta 分析（随机效应）

## ================================

meta_res <- metacont(

n.e = n_e, mean.e = mean_e, sd.e = sd_e,

n.c = n_c, mean.c = mean_c, sd.c = sd_c,

studlab = Study,

data = data,

sm = "SMD",

method.smd = "Hedges",

method.tau = "REML",

method.tau.ci = "J",

comb.random = TRUE,

comb.fixed = FALSE,

prediction = TRUE

)

## ================================

## 3. 配色：渐变蓝

## ================================

pal_fn <- grDevices::colorRampPalette(c("#6BAED6", "#3182BD", "#08519C"))

pal <- pal_fn(200)

col_line <- "#0B3C5D"

map_to_col <- function(x, pal, rng = NULL) {

if (is.null(rng)) rng <- range(x, na.rm = TRUE)

if (!is.finite(diff(rng)) || diff(rng) == 0) return(rep(pal[length(pal)], length(x)))

idx <- floor((x - rng[1]) / diff(rng) * (length(pal) - 1)) + 1

pal[pmax(1, pmin(length(pal), idx))]

}

te_rng <- range(meta_res$TE, na.rm = TRUE)

col_sq_vec <- map_to_col(meta_res$TE, pal, rng = te_rng)

col_predict <- grDevices::adjustcolor(col_line, alpha.f = 0.35)

col_predict_lines <- grDevices::adjustcolor(col_line, alpha.f = 0.70)

## ================================

## 4. 绘制森林图：显示 Test for overall effect + 防挤压

## ================================

forest(

meta_res,

plotwidth = "13cm",

leftcols = c("studlab"),

rightcols = c("effect", "ci", "w.random"),

rightlabs = c("Hedge's g", "95% CI", "Weight"),

col.square = col_sq_vec,

col.square.lines = col_line,

col.study = col_sq_vec,

col.diamond = col_line,

col.diamond.lines = col_line,

col.predict = col_predict,

col.predict.lines = col_predict_lines,

fontsize = 9,

spacing = 1,

fs.hetstat = 9,

fs.axis = 9,

prediction = TRUE,

digits = 2,

print.tau2 = TRUE,

print.tau2.ci = TRUE,

print.tau = TRUE,

test.overall.random = TRUE,

addrows.below.overall = 2,

xlab = "Hedge's g"

)
