# Supplementary material for: Does resistance training alone or in combination with aerobic training improve vascular function indices in adults with type 2 diabetes? A systematic review and meta-analysis of randomized controlled trials
Source: Front Endocrinol (Lausanne). 2026 May 15;17:1824213. doi: 10.3389/fendo.2026.1824213 (PMC13218868; doi:10.3389/fendo.2026.1824213)

| Study | Experiment | | | Control | | |
| --- | --- | --- | --- | --- | --- | --- |
|  | Total | MEAN | SD | Total | MEAN | SD |
| Magalhães et al., 2019 (crPWV) | 28 | 9.0 | 2.1 | 27 | 9.3 | 1.5 |
| Magalhães et al., 2019 (crPWV) | 25 | 8.3 | 1.9 | 27 | 9.3 | 1.5 |

## ================================

## 0. 环境准备

## ================================

library(meta)

## ================================

## 1. 构建数据（已替换为 crPWV.docx 中的数据）

## ================================

data <- data.frame(

Study = c(

"Magalhães et al., 2019 (crPWV)",

"Magalhães et al., 2019 (crPWV)"

),

n_e = c(28, 25),

mean_e = c(9.0, 8.3),

sd_e = c(2.1, 1.9),

n_c = c(27, 27),

mean_c = c(9.3, 9.3),

sd_c = c(1.5, 1.5)

)

## ================================

## 2. Meta 分析（随机效应）

## ================================

meta_res <- metacont(

n.e = n_e, mean.e = mean_e, sd.e = sd_e,

n.c = n_c, mean.c = mean_c, sd.c = sd_c,

studlab = Study,

data = data,

sm = "SMD",

method.smd = "Hedges",

method.tau = "REML",

method.tau.ci = "J",

comb.random = TRUE,

comb.fixed = FALSE,

prediction = TRUE

)

## ================================

## 3. 配色：渐变蓝

## ================================

pal_fn <- grDevices::colorRampPalette(c("#6BAED6", "#3182BD", "#08519C"))

pal <- pal_fn(200)

col_line <- "#0B3C5D"

map_to_col <- function(x, pal, rng = NULL) {

if (is.null(rng)) rng <- range(x, na.rm = TRUE)

if (!is.finite(diff(rng)) || diff(rng) == 0) return(rep(pal[length(pal)], length(x)))

idx <- floor((x - rng[1]) / diff(rng) * (length(pal) - 1)) + 1

pal[pmax(1, pmin(length(pal), idx))]

}

te_rng <- range(meta_res$TE, na.rm = TRUE)

col_sq_vec <- map_to_col(meta_res$TE, pal, rng = te_rng)

col_predict <- grDevices::adjustcolor(col_line, alpha.f = 0.35)

col_predict_lines <- grDevices::adjustcolor(col_line, alpha.f = 0.70)

## ================================

## 4. 绘制森林图：显示 Test for overall effect + 防挤压

## ================================

forest(

meta_res,

plotwidth = "13cm",

leftcols = c("studlab"),

rightcols = c("effect", "ci", "w.random"),

rightlabs = c("Hedge's g", "95% CI", "Weight"),

col.square = col_sq_vec,

col.square.lines = col_line,

col.study = col_sq_vec,

col.diamond = col_line,

col.diamond.lines = col_line,

col.predict = col_predict,

col.predict.lines = col_predict_lines,

fontsize = 9,

spacing = 1,

fs.hetstat = 9,

fs.axis = 9,

prediction = TRUE,

digits = 2,

print.tau2 = TRUE,

print.tau2.ci = TRUE,

print.tau = TRUE,

## ✅ 关键1：直接让 forest 打印 overall effect 的 Z 与 p（随机效应）

test.overall.random = TRUE,

## ✅ 关键2：在“总体结果”和“异质性/检验信息(x轴下方)”之间加空行，避免挤在一起

addrows.below.overall = 2,

## x轴标题直接用 forest 的 xlab（比 mtext 稳）

xlab = "Hedge's g"

)

## ================================

## 2.1 查看完整统计结果（含Q等）

## ================================

print(summary(meta_res))

## ================================

## 2.2 提取 Q + 计算 Q-test Power(%)

## （基于观察到的Q的事后/近似 achieved power）

## ================================

Q_val <- meta_res$Q

df_Q <- meta_res$df.Q

p_Q <- meta_res$pval.Q

alpha_Q <- 0.10 # 常用于Q异质性检验；如需0.05改这里

Q_crit <- qchisq(1 - alpha_Q, df = df_Q)

## 非中心参数常用近似：lambda ≈ max(0, Q - df)

lambda <- max(0, Q_val - df_Q)

Power_Qtest_pct <- 100 * (1 - pchisq(Q_crit, df = df_Q, ncp = lambda))

out_Q_power <- data.frame(

Q = Q_val,

df = df_Q,

p_Q = p_Q,

alpha = alpha_Q,

Q_crit = Q_crit,

lambda = lambda,

Power_Qtest_pct = Power_Qtest_pct

)

print(out_Q_power)

## 如果你只要两项（Q 和 Power%），用这个：

Q_and_Power <- data.frame(

Q = Q_val,

Power_Qtest_pct = Power_Qtest_pct

)

print(Q_and_Power)


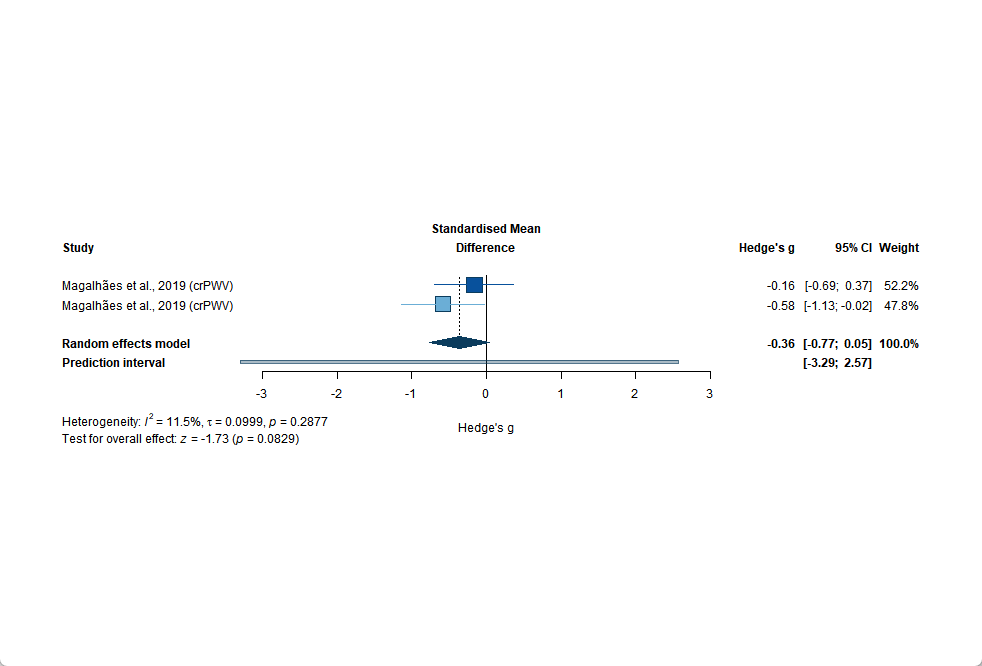

Supplement: Supplementary file 1 [file DataSheet1.zip › Supplementary File/Arterial stiffness/Subgroup analysis/Arterial stiffness measure type/crPWV.docx]
