# Supplementary material for: Does resistance training alone or in combination with aerobic training improve vascular function indices in adults with type 2 diabetes? A systematic review and meta-analysis of randomized controlled trials
Source: Front Endocrinol (Lausanne). 2026 May 15;17:1824213. doi: 10.3389/fendo.2026.1824213 (PMC13218868; doi:10.3389/fendo.2026.1824213)
Supplement: Supplementary file 1 [file DataSheet1.zip › Supplementary File/Arterial stiffness/Subgroup analysis/Arterial stiffness measure type/Subgroup .docx]

| Subgroup | Hedges’g | 95% CI |
| --- | --- | --- |
| PWV | -0.21 | -0.56 to 0.13 |
| cfPWV | -0.06 | -0.28 to 0.15 |
| cdPWV | -0.63 | -1.15 to -0.12 |
| crPWV | -0.36 | -0.77 to 0.05 |
| β-stiffness index | -0.31 | -0.69 to 0.07 |

## ================================

## 0. 环境准备

## ================================

library(meta)

## ================================

## 1. 构建数据（来自 亚组.docx：Hedge's g + 95% CI）

## ================================

data <- data.frame(

Study = c(

"PWV",

"cfPWV",

"cdPWV",

"crPWV",

"β-stiffness index"

),

TE = c(-0.21, -0.06, -0.63, -0.36, -0.31),

lower = c(-0.56, -0.28, -1.15, -0.77, -0.69),

upper = c( 0.13, 0.15, -0.12, 0.05, 0.07)

)

## 由 95% CI 反推标准误 seTE：se ≈ (upper - lower) / (2*1.96)

data$seTE <- (data$upper - data$lower) / (2 * 1.96)

## ================================

## 2. Meta 分析（随机效应）

## ================================

meta_res <- metagen(

TE = TE,

seTE = seTE,

studlab = Study,

data = data,

sm = "SMD",

method.tau = "REML",

method.tau.ci = "J",

comb.random = TRUE,

comb.fixed = FALSE,

prediction = TRUE

)

## ================================

## 3. 配色：渐变蓝

## ================================

pal_fn <- grDevices::colorRampPalette(c("#6BAED6", "#3182BD", "#08519C"))

pal <- pal_fn(200)

col_line <- "#0B3C5D"

map_to_col <- function(x, pal, rng = NULL) {

if (is.null(rng)) rng <- range(x, na.rm = TRUE)

if (!is.finite(diff(rng)) || diff(rng) == 0) return(rep(pal[length(pal)], length(x)))

idx <- floor((x - rng[1]) / diff(rng) * (length(pal) - 1)) + 1

pal[pmax(1, pmin(length(pal), idx))]

}

te_rng <- range(meta_res$TE, na.rm = TRUE)

col_sq_vec <- map_to_col(meta_res$TE, pal, rng = te_rng)

col_predict <- grDevices::adjustcolor(col_line, alpha.f = 0.35)

col_predict_lines <- grDevices::adjustcolor(col_line, alpha.f = 0.70)

## ================================

## 4. 绘制森林图：显示 Test for overall effect + 防挤压

## ================================

forest(

meta_res,

plotwidth = "13cm",

leftcols = c("studlab"),

rightcols = c("effect", "ci", "w.random"),

rightlabs = c("Hedge's g", "95% CI", "Weight"),

col.square = col_sq_vec,

col.square.lines = col_line,

col.study = col_sq_vec,

col.diamond = col_line,

col.diamond.lines = col_line,

col.predict = col_predict,

col.predict.lines = col_predict_lines,

fontsize = 9,

spacing = 1,

fs.hetstat = 9,

fs.axis = 9,

prediction = TRUE,

digits = 2,

print.tau2 = TRUE,

print.tau2.ci = TRUE,

print.tau = TRUE,

test.overall.random = TRUE,

addrows.below.overall = 2,

xlab = "Hedge's g"

)
