# Supplementary material for: Does resistance training alone or in combination with aerobic training improve vascular function indices in adults with type 2 diabetes? A systematic review and meta-analysis of randomized controlled trials
Source: Front Endocrinol (Lausanne). 2026 May 15;17:1824213. doi: 10.3389/fendo.2026.1824213 (PMC13218868; doi:10.3389/fendo.2026.1824213)

| X：Repetitions | Y：（effect size）Hedge's g | Weight（%） |
| --- | --- | --- |
| 10-15 | 0.66 | 10.2 |
| to failure | 1.83 | 9.1 |
| 10-25 | 0.43 | 15.4 |
| 10 | 0.41 | 15.4 |

# 加载必要的包

library(metafor)

# ================================

# 1) 用 Repetitions.docx 的数据替换

# ================================

df <- data.frame(

Repetitions = c("10-15", "to failure", "10-25", "10"),

g = c(0.66, 1.83, 0.43, 0.41),

Weight = c(10.2, 9.1, 15.4, 15.4)

)

# 计算方差（权重为 1/vi）

df$vi <- 1 / df$Weight

# ================================

# 2) 将 Repetitions 转为数值（区间取中点；to failure -> NA）

# ================================

df$Reps_num <- sapply(df$Repetitions, function(x) {

x0 <- tolower(trimws(x))

if (grepl("failure", x0)) return(NA_real_) # "to failure" 无法直接数值化

if (grepl("-", x0)) {

p <- as.numeric(strsplit(x0, "-", fixed = TRUE)[[1]])

return(mean(p))

}

as.numeric(x0)

})

# 连续型 meta 回归：自动剔除 Reps_num 为 NA 的行（即 to failure）

df_cont <- subset(df, !is.na(Reps_num))

# ================================

# 3) 执行 Meta 回归分析（混合效应模型）

# ================================

res <- rma(yi = g, vi = vi, mods = ~ Reps_num, data = df_cont)

# 提取统计结果（稳健写法）

tab <- coef(summary(res)) # estimate, se, zval, pval, ci.lb, ci.ub

beta <- round(tab[2, "estimate"], 3)

ci_lb <- round(tab[2, "ci.lb"], 3)

ci_ub <- round(tab[2, "ci.ub"], 3)

p_value <- ifelse(tab[2, "pval"] < 0.001, "< 0.001", round(tab[2, "pval"], 3))

# ================================

# 4) 绘制气泡图（连续型：Reps_num）

# ================================

regplot(

res,

mod = "Reps_num",

pi = TRUE,

pred = TRUE,

xlab = "Repetitions",

ylab = "Hedge's g",

psize = sqrt(df_cont$Weight),

col = "black",

ci.col = "darkgray",

pi.col = "lightgray",

las = 1

)

# 添加统计结果文本

text(

x = max(df_cont$Reps_num) - 0.2 * (max(df_cont$Reps_num) - min(df_cont$Reps_num)),

y = max(df_cont$g) - 0.1 * (max(df_cont$g) - min(df_cont$g)),

labels = paste0(

"β=", beta, "\n",

"95% CI: [", ci_lb, ", ", ci_ub, "]\n",

"P=", p_value

),

pos = 2,

cex = 0.9,

col = "black",

font = 2

)

# 添加紧凑图例

legend(

"bottomright",

legend = c("Studies", "Regression Line", "95% Confidence Interval", "95% Prediction Interval"),

pch = c(19, NA, NA, NA),

lty = c(NA, 1, NA, NA),

fill = c(NA, NA, "darkgray", "lightgray"),

border = c(NA, NA, "darkgray", "lightgray"),

col = c("gray60", "black", NA, NA),

pt.cex = 1.0,

cex = 0.62,

x.intersp = 0.75,

y.intersp = 0.75,

bg = "white"

)

# ================================

# 备选方案（可选）：把 “to failure” 当作二分类变量纳入所有研究

# ================================

# df$to_failure <- ifelse(grepl("failure", tolower(df$Repetitions)), 1, 0)

# res2 <- rma(yi = g, vi = vi, mods = ~ to_failure, data = df)

# regplot(

# res2,

# mod = "to_failure",

# pi = TRUE,

# pred = TRUE,

# xlab = "To failure (0=No, 1=Yes)",

# ylab = "Hedge's g",

# psize = sqrt(df$Weight),

# col = "black",

# ci.col = "darkgray",

# pi.col = "lightgray",

# las = 1

# )


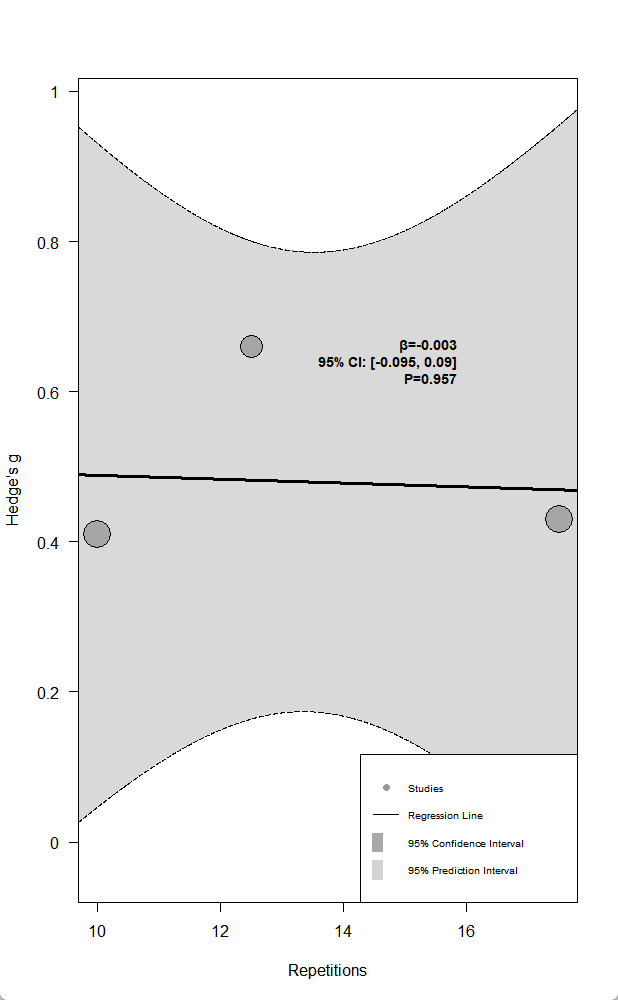

Supplement: Supplementary file 1 [file DataSheet1.zip › Supplementary File/FMD/Meta-regression analysis/Repetitions.docx]
