# Supplementary material for: Does resistance training alone or in combination with aerobic training improve vascular function indices in adults with type 2 diabetes? A systematic review and meta-analysis of randomized controlled trials
Source: Front Endocrinol (Lausanne). 2026 May 15;17:1824213. doi: 10.3389/fendo.2026.1824213 (PMC13218868; doi:10.3389/fendo.2026.1824213)

| X：Time(min) | Y：（effect size）Hedge's g | Weight（%） |
| --- | --- | --- |
| 3 | 0.56 | 15.6 |
| 6 | 0.22 | 16.0 |
| 40 | 0.66 | 10.2 |
| 75 | 0.60 | 13.2 |
| 60 | 1.83 | 9.1 |
| 60 | 0.85 | 5.1 |
| 26 | 0.43 | 15.4 |
| 52.5 | 0.41 | 15.4 |

# 加载必要的包

library(metafor)

# 创建数据框（来自 Time(min).docx）

df <- data.frame(

Time = c(3, 6, 40, 75, 60, 60, 26, 52.5),

g = c(0.56, 0.22, 0.66, 0.60, 1.83, 0.85, 0.43, 0.41),

Weight = c(15.6, 16.0, 10.2, 13.2, 9.1, 5.1, 15.4, 15.4)

)

# 计算方差（权重为1/vi）

df$vi <- 1 / df$Weight

# 执行Meta回归分析（混合效应模型）

res <- rma(yi = g, vi = vi, mods = ~ Time, data = df)

# 提取统计结果（稳健写法：从 summary(res) 的系数表取数值）

tab <- coef(summary(res)) # estimate, se, zval, pval, ci.lb, ci.ub

beta <- round(tab[2, "estimate"], 3)

ci_lb <- round(tab[2, "ci.lb"], 3)

ci_ub <- round(tab[2, "ci.ub"], 3)

p_value <- ifelse(tab[2, "pval"] < 0.001, "< 0.001", round(tab[2, "pval"], 3))

# 绘制气泡图

regplot(

res,

mod = "Time",

pi = TRUE,

pred = TRUE,

xlab = "Time (min)",

ylab = "Hedge's g",

psize = sqrt(df$Weight),

col = "black",

ci.col = "darkgray",

pi.col = "lightgray",

las = 1

)

# 添加统计结果文本

text(

x = max(df$Time) - 0.2*(max(df$Time) - min(df$Time)), # x位置：右端偏左20%

y = max(df$g) - 0.1*(max(df$g) - min(df$g)), # y位置：顶端偏下10%

labels = paste0(

"β=", beta, "\n",

"95% CI: [", ci_lb, ", ", ci_ub, "]\n",

"P=", p_value

),

pos = 2,

cex = 0.9,

col = "black",

font = 2

)

# 添加紧凑图例

legend(

"bottomright",

legend = c("Studies", "Regression Line", "95% Confidence Interval", "95% Prediction Interval"),

pch = c(19, NA, NA, NA),

lty = c(NA, 1, NA, NA),

fill = c(NA, NA, "darkgray", "lightgray"),

border = c(NA, NA, "darkgray", "lightgray"),

col = c("gray60", "black", NA, NA),

pt.cex = 1.0,

cex = 0.62,

x.intersp = 0.75,

y.intersp = 0.75,

bg = "white"

)


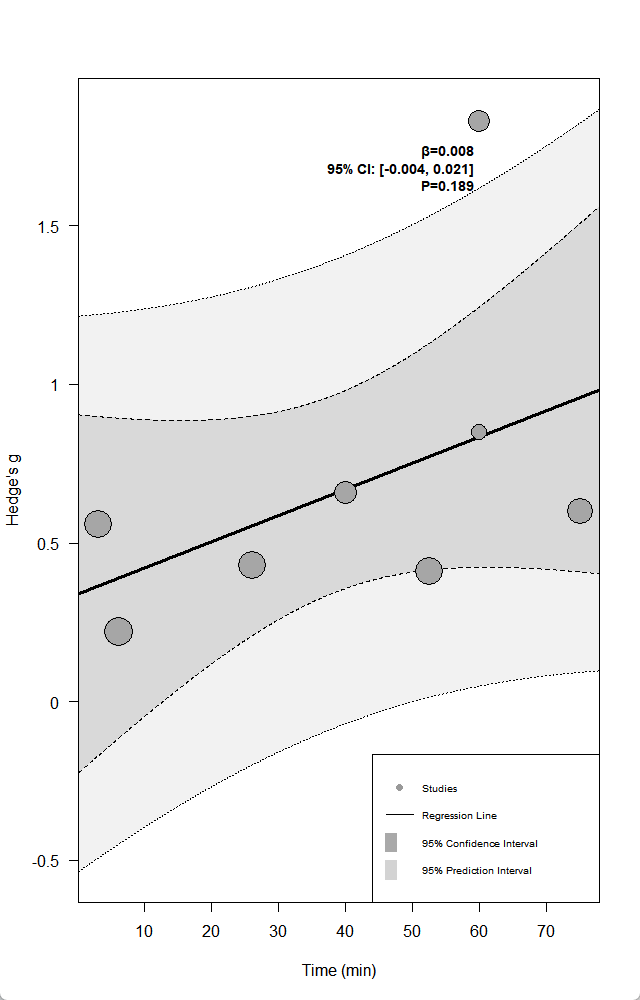

Supplement: Supplementary file 1 [file DataSheet1.zip › Supplementary File/FMD/Meta-regression analysis/Time(min).docx]
