# Supplementary material for: Does resistance training alone or in combination with aerobic training improve vascular function indices in adults with type 2 diabetes? A systematic review and meta-analysis of randomized controlled trials
Source: Front Endocrinol (Lausanne). 2026 May 15;17:1824213. doi: 10.3389/fendo.2026.1824213 (PMC13218868; doi:10.3389/fendo.2026.1824213)
Supplement: Supplementary file 1 [file DataSheet1.zip › Supplementary File/FMD/Subgroup analysis/Age/Subgroup .docx]

| Subgroup |  | Hedge's g | 95% CI |
| --- | --- | --- | --- |
| Young Adult: 19–24 | 1(13) | 0.85 | -0.33 to 2.03 |
| Middle Aged: 45–64 | 7(285) | 0.60 | 0.29 to 0.90 |

## ================================

## 0. 环境准备

## ================================

library(meta)

## ================================

## 1. 构建数据（来自：亚组.docx）

## ================================

data <- data.frame(

Subgroup = c(

"Young Adult: 19–24",

"Middle Aged: 45–64"

),

Studies_Participants = c("1(13)", "7(285)"),

g = c(0.85, 0.60),

lower = c(-0.33, 0.29),

upper = c(2.03, 0.90)

)

## 由95%CI反推标准误：SE = (upper - lower) / (2*1.96)

data$se <- (data$upper - data$lower) / (2 * 1.96)

## ================================

## 2. Meta 分析（随机效应；基于 g + SE）

## ================================

meta_res <- metagen(

TE = g,

seTE = se,

studlab = Subgroup,

data = data,

sm = "SMD",

method.tau = "REML",

method.tau.ci = "J",

comb.random = TRUE,

comb.fixed = FALSE,

prediction = TRUE

)

## ================================

## 3. 配色：渐变蓝

## ================================

pal_fn <- grDevices::colorRampPalette(c("#6BAED6", "#3182BD", "#08519C"))

pal <- pal_fn(200)

col_line <- "#0B3C5D"

map_to_col <- function(x, pal, rng = NULL) {

if (is.null(rng)) rng <- range(x, na.rm = TRUE)

if (!is.finite(diff(rng)) || diff(rng) == 0) return(rep(pal[length(pal)], length(x)))

idx <- floor((x - rng[1]) / diff(rng) * (length(pal) - 1)) + 1

pal[pmax(1, pmin(length(pal), idx))]

}

te_rng <- range(meta_res$TE, na.rm = TRUE)

col_sq_vec <- map_to_col(meta_res$TE, pal, rng = te_rng)

col_predict <- grDevices::adjustcolor(col_line, alpha.f = 0.35)

col_predict_lines <- grDevices::adjustcolor(col_line, alpha.f = 0.70)

## ================================

## 4. 绘制森林图：显示 overall effect + 防挤压

## ================================

forest(

meta_res,

plotwidth = "13cm",

leftcols = c("studlab", "Studies_Participants"),

leftlabs = c("Subgroup", "k(N)"),

rightcols = c("effect", "ci", "w.random"),

rightlabs = c("Hedge's g", "95% CI", "Weight"),

col.square = col_sq_vec,

col.square.lines = col_line,

col.study = col_sq_vec,

col.diamond = col_line,

col.diamond.lines = col_line,

col.predict = col_predict,

col.predict.lines = col_predict_lines,

fontsize = 9,

spacing = 1,

fs.hetstat = 9,

fs.axis = 9,

prediction = TRUE,

digits = 2,

print.tau2 = TRUE,

print.tau2.ci = TRUE,

print.tau = TRUE,

test.overall.random = TRUE,

addrows.below.overall = 2,

xlab = "Hedge's g"

)
