# Supplementary material for: Does resistance training alone or in combination with aerobic training improve vascular function indices in adults with type 2 diabetes? A systematic review and meta-analysis of randomized controlled trials
Source: Front Endocrinol (Lausanne). 2026 May 15;17:1824213. doi: 10.3389/fendo.2026.1824213 (PMC13218868; doi:10.3389/fendo.2026.1824213)
Supplement: Supplementary file 1 [file DataSheet1.zip › Supplementary File/FMD/Subgroup analysis/Sets/Subgroup .docx]

| Subgroup | Hedge's g | 95% CI |
| --- | --- | --- |
| 3 | 0.66 | -0.05 to 1.26 |
| 8 | 0.42 | 0.00 to 0.83 |
