# Supplementary material for: Does resistance training alone or in combination with aerobic training improve vascular function indices in adults with type 2 diabetes? A systematic review and meta-analysis of randomized controlled trials
Source: Front Endocrinol (Lausanne). 2026 May 15;17:1824213. doi: 10.3389/fendo.2026.1824213 (PMC13218868; doi:10.3389/fendo.2026.1824213)

| Study | Experiment | | | Control | | |
| --- | --- | --- | --- | --- | --- | --- |
|  | Total | MEAN | SD | Total | MEAN | SD |
| Kwon et al., 2011 | 12 | 5.6 | 2.8 | 15 | 4.0 | 1.9 |
| Cox et al., 2024 | 23 | 4.4 | 2.5 | 23 | 3.5 | 1.8 |

## ================================

## 0. 环境准备

## ================================

library(meta)

## ================================

## 1. 构建数据（替换为 27-59.docx）

## ================================

data <- data.frame(

Study = c(

"Kwon et al., 2011",

"Cox et al., 2024"

),

n_e = c(12, 23),

mean_e = c(5.6, 4.4),

sd_e = c(2.8, 2.5),

n_c = c(15, 23),

mean_c = c(4.0, 3.5),

sd_c = c(1.9, 1.8)

)

## ================================

## 2. Meta 分析（随机效应）

## ================================

meta_res <- metacont(

n.e = n_e, mean.e = mean_e, sd.e = sd_e,

n.c = n_c, mean.c = mean_c, sd.c = sd_c,

studlab = Study,

data = data,

sm = "SMD",

method.smd = "Hedges",

method.tau = "REML",

method.tau.ci = "J",

comb.random = TRUE,

comb.fixed = FALSE,

prediction = TRUE

)

## ================================

## 3. 配色：渐变蓝

## ================================

pal_fn <- grDevices::colorRampPalette(c("#6BAED6", "#3182BD", "#08519C"))

pal <- pal_fn(200)

col_line <- "#0B3C5D"

map_to_col <- function(x, pal, rng = NULL) {

if (is.null(rng)) rng <- range(x, na.rm = TRUE)

if (!is.finite(diff(rng)) || diff(rng) == 0) return(rep(pal[length(pal)], length(x)))

idx <- floor((x - rng[1]) / diff(rng) * (length(pal) - 1)) + 1

pal[pmax(1, pmin(length(pal), idx))]

}

te_rng <- range(meta_res$TE, na.rm = TRUE)

col_sq_vec <- map_to_col(meta_res$TE, pal, rng = te_rng)

col_predict <- grDevices::adjustcolor(col_line, alpha.f = 0.35)

col_predict_lines <- grDevices::adjustcolor(col_line, alpha.f = 0.70)

## ================================

## 4. 绘制森林图：显示 Test for overall effect + 防挤压

## ================================

forest(

meta_res,

plotwidth = "13cm",

leftcols = c("studlab"),

rightcols = c("effect", "ci", "w.random"),

rightlabs = c("Hedge's g", "95% CI", "Weight"),

col.square = col_sq_vec,

col.square.lines = col_line,

col.study = col_sq_vec,

col.diamond = col_line,

col.diamond.lines = col_line,

col.predict = col_predict,

col.predict.lines = col_predict_lines,

fontsize = 9,

spacing = 1,

fs.hetstat = 9,

fs.axis = 9,

prediction = TRUE,

digits = 2,

print.tau2 = TRUE,

print.tau2.ci = TRUE,

print.tau = TRUE,

test.overall.random = TRUE,

addrows.below.overall = 2,

xlab = "Hedge's g"

)


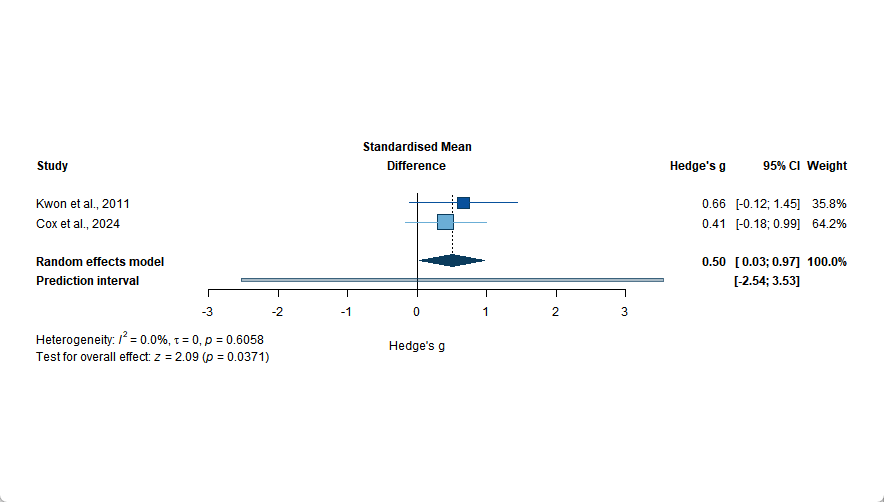

Supplement: Supplementary file 1 [file DataSheet1.zip › Supplementary File/FMD/Subgroup analysis/Time(min)/27-59.docx]
